# Design of a Novel Multi-Epitope Vaccine Against *Echinococcus granulosus* in Immunoinformatics

**DOI:** 10.3389/fimmu.2021.668492

**Published:** 2021-08-12

**Authors:** Mingkai Yu, Yuejie Zhu, Yujiao Li, Zhiqiang Chen, Tong Sha, Zhiwei Li, Fengbo Zhang, Jianbing Ding

**Affiliations:** ^1^Department of Immunology, School of Basic Medical Sciences, Xinjiang Medical University, Urumqi, China; ^2^Reproductive Medicine Center, The First Affiliated Hospital of Xinjiang Medical University, Urumqi, China; ^3^Department of Blood Transfusion, The First Affiliated Hospital of Xinjiang Medical University, Urumqi, China; ^4^Clinical Laboratory Center, Xinjiang Uygur Autonomous Region People's Hospital, Urumqi, China; ^5^Department of Clinical Laboratory, The First Affiliated Hospital of Xinjiang Medical University, Urumqi, China; ^6^State Key Laboratory of Pathogenesis, Prevention, Treatment of Central Asian High Incidence Diseases, The First Affiliated Hospital of Xinjiang Medical University, Urumqi, China

**Keywords:** immunoinformatics, multi-epitope, *Echinococcus granulosus*, molecular docking, vaccine

## Abstract

All the time, echinococcosis is a global zoonotic disease which seriously endangers public health all over the world. In order to speed up the development process of anti-*Echinococcus granulosus* vaccine, at the same time, it can also save economic cost. In this study, immunoinformatics tools and molecular docking methods were used to predict and screen the antigen epitopes of *Echinococcus granulosus*, to design a multi-epitope vaccine containing B- and T-cell epitopes. The multi-epitope vaccine could activate B lymphocytes to produce specific antibodies theoretically, which could protect the human body against *Echinococcus granulosus* infection. It also could activate T lymphocytes and clear the infected parasites in the body. In this study, four CD8^+^ T-cell epitopes, three CD4^+^ T-cell epitopes and four B-cell epitopes of Protein EgTeg were identified by immunoinformatics methods. Meanwhile, three CD8^+^ T-cell epitopes, two CD4^+^ T-cell epitopes and four B-cell epitopes of Protein EgFABP1 were identified. We constructed the multi-epitope vaccine using linker proteins. The study based on the traditional methods of antigen epitope prediction, further optimized the prediction results combined with molecular docking technology and improved the precision and accuracy of the results. Finally, *in vivo* and *in vitro* experiments had verified that the vaccine designed in this study had good antigenicity and immunogenicity.

## Introduction

Cystic echinococcosis (CE) or cystic hydatid disease (CHD) is a worldwide spread zoonotic disease caused by the larval stage of the *Echinococcus granulosus* (*E. g*) tapeworm. The human CE takes on a worldwide distribution and the annual incidence as high as (1-200)/100,000 in endemic areas (1). The World Organization for Animal Health (OIE) listed it as the class B animal epidemic and the World Health Organization (WHO) listed it as one of the 17 diseases to be controlled or eliminated in 2050, so it can be seen that echinococcosis has become a serious social problem which endangering public health (2). In China, it belongs to the class II of animal epidemic. The first Chinese epidemiological survey of CE indicated that the prevalence range of disease is wide, the self-protection awareness of the crowd is poor, the propaganda and control power of disease is weak and the number of infected persons and the burden of social treatment ranked firstly in the world for China (3, 4). *E. g* mainly parasitizes in the liver and lung and easily to form a cyst even endanger life, with high fatality rate (5, 6). The treatment choice of CE is complex, as the various clinical manifestations of this disease (7). In patients, current therapy options include surgical procedures, percutaneous therapy and chemotherapy. Despite the wide use of these therapeutic methods, CE is still prevalent globally (8, 9). Moreover, the poor prognoses and non-effective diagnosis have led researches towards echinococcosis prevention strategies. The search for anti-hydatid vaccine will become the most effective means to prevent echinococcosis undoubtedly (10). Traditional vaccine development is a costly and time-consuming procedure, particularly for CE with the complex life cycle.

With the rapid development of molecular biology, a large number of genomics and proteomics biological databases could provide more targets for construction of vaccines, nowadays researchers have screened out a series of candidate protein for echinococcosis vaccine (11). The Protein EgTeg is a candidate vaccine protein that Ortona et al. (12) used the serum’s IgG4 in patients with active cystic echinococcosis to screened of a cDNA library, a cDNA that encodes partial amino acids in larval stage was isolated from this library and the protein was found in the *E. g*’s protoscolex and cyst wall by immunofluorescent antibody technique. This protein can inhibit the chemotaxis of neutrophils significantly in the early stage of infection and it is a tegumental protein probably related to parasite survival (13). The Protein EgFABP1 was already confirmed that can be expressed at the protoscolex and on the whole life of *E. g.* It is a complete protective antigen and effects on the absorption of essential fatty acids for growth of parasites mainly. In addition, it can stimulate the body to produce specific antibody IgE (14).

In this study, we used different immunoinformatics methods to analyze the structures and physicochemical parameters of proteins EgTeg and EgFABP1. Concurrently, we predicted the T-cell epitopes (TEs) and B-cell epitopes (BEs) that combined of different prediction software. The efficient antigens should have good antigenicity and immunogenicity, it can induce body’s specific immune response. But the function of peptide vaccine that was made by single epitope is too weak to meet the standards of vaccine application actually (15). Linker protein is a kind of short peptide that links different target proteins in the gene fusion technology (16), it can improve the immunogenicity by linking different antigen epitopes into one, thus we made a fusion protein that was composed of epitopes of EgTeg and EgFABP1. We used the linker protein to construct the peptide vaccine preliminarily and *in vitro* and *in vivo* experiments were carried out to verified to test its effectiveness. The current study can provide theories and data for further research, increase a new method and idea for the development of multivalent epitope vaccine of echinococcosis (17).

## Materials and Methods

### The Amino Acid Sequences of Proteins 

The online database of National Center for Biotechnology Information (NCBI) (https://www.ncbi.nlm.nih.gov/genbank/)was used to obtain the amino acid sequences of Protein EgTeg and EgFABP1.

### The Prediction of Physicochemical Parameters of Proteins 

The online software ProtParam (http://web.expasy.org/protparam/) was used to analyze the physicochemical parameters of Protein EgTeg and EgFABP1, including atomic composition, molecular weight, theoretical isoelectric point (PI), charged polar, stability, hydrophobicity and so on.

### The Prediction of Transmembrane Domains and Signal Peptide of Proteins 

The online server TMHMM (http://www.cbs.dtu.dk/services/TMHMM-2.0/) was used to analyze the transmembrane domains of Protein EgTeg and EgFABP1. The online server signalP-5.0 (http://www.cbs.dtu.dk/services/SignalP-5.0/) was used to analyze the signal peptide.

### The Prediction of Phosphorylation Sites of Proteins

The online server NetPhos3.1 (http://www.cbs.dtu.dk/services/NetPhos/) was used to analyze the phosphorylation sites of Protein EgTeg and EgFABP1.

### The Prediction of Secondary Structure of Proteins

The secondary structure of Protein EgTeg and EgFABP1 was analyzed by software DNASTAR and online software SOMPA (https://npsaprabi.ibcp.fr/cgibin/npsa_automat.pl?page=npsa_sopma.html), what was analyzed includes Alpha helix, Extended strand, Beta turn and Random coil.

### The Prediction of Tertiary Structure of Proteins

Firstly, the online software SWISS-MODEL (https://www.swissmodel.expasy.org/interactive) was applied to find a high homology model with Protein EgTeg for predicting exactly, but the homology between all the models found and Protein EgTeg lower than 30%. Then the tertiary structure of Protein EgTeg was reanalyzed by I-TASSER (https://zhanglab.ccmb.med.umich.edu/I-TASSER/), meanwhile the software Discover Studio (DS) was used to view the tertiary structure and then the Ramachandran plot was made to evaluate the rationality. The tertiary structure of Protein EgFABP1 has been determined by Jakobsson et al. (18), we obtained the result from database NCBI (https://www.ncbi.nlm.nih.gov/Structure/mmdb/mmdbsrv.cgi?dps=0&uid=1O8V).

### The Prediction of T-Cell Epitopes of Proteins

T-cell antigen receptor (TCR) is a specific receptor for T-cell to recognize antigen, the antigen type of major histocompatibility complex (MHC) on antigen presenting cells (APC) must be recognized firstly when TCR recognizes antigenic peptide, so the T-cell epitopes were divided into CD8^+^ and CD4^+^ two types. The MHC was called human leukocyte antigen (HLA) in humans, according to a previous study, the HLA-A*1101 (13.46%), HLA-A*0201 (12.50%), and HLA-A*0301 (10.10%), HLA-DRB1*0701 (16.35%), HLA-DRB1*1501 (8.65%) and HLA-DRB1*0301 (7.69%) are the high frequency HLA alleles in Xinjiang of China (19). The online software SYFPEITHI (http://www.syfpeithi.de/bin/mhcserver.dll/epitopeprediction.htm) and IEDB (http://tools.iedb.org/mhci/) were used for analyzing the T-cell epitopes. Finally, we combined the results as the final TE of two software for high accuracy.

### The Prediction of B-Cell Epitopes of Proteins

The Linear epitope, β-turn, Surface accessibility, Flexibility, Antigenicity and Hydrophilicity of Protein EgTeg and EgFABP1 was analyzed by online software IEDB (http://tools.immuneepitope.org/main/bcell/) and then the Surface accessibility, Antigenicity, Flexibility and Hydrophilicity were analyzed by software DNASTAR. These regions are easy to form epitopes, so we took the common parts of these domains to define as the B-cell epitopes (BE). We combined the results from the two software to be the final BE.

### The Optimization of CD4^+^ T-Cell Epitopes 

In the human immune response, the HLA-class II molecule need bind to antigen peptide and then deliver it to TCR for activating T-cell. By molecule docking between antigen peptide and HLA-II, the CD4^+^ T-cell epitopes could be further optimized. For getting the crystalline structure of HLA-II class alleles, we obtained a PDB format complex that was HLA-II class molecule bound with a 14aa short peptide from NCBI structure database (20). We first knocked out the short peptide in this complex, the residual structure was HLA-DRB1 and it belonged to HLA-class II alleles. Then the CD4^+^ T-cell epitopes predict-ed previously was docked with the HLA-DRB1 using software DS, we selected the best docking result to conduct RDOCK process of software DS and then the residues that participated in the interaction was found by calculating the Root Mean Squared Deviation (RMSD) of binding site, we further optimized out new CD4^+^ T-cell epitopes.

### The Construction of Multi-Epitope Peptide Vaccine

We accorded to the research of Pourseif et al. (21), some linker proteins were selected to construct our vaccine model. The CD8^+^ T-cell, optimized CD4^+^ T-cell and B-cell epitopes of EgTeg and EgFABP1 were connected by Linker-GPSL one by one. In this vaccine construction, there are two repeats of optimized CD4^+^ T-cell epitopes for obtaining maximal immune system responses. And in the two repeats of CD4^+^ T-cell epitopes, we selected Linker-HEYGAEALERAG to link the two same copies. Between of T-cell epitopes was Linker-KFERQ and between of B-cell epitopes was Linker-GGSSGG. Later, we constructed the 3D structure of the vaccine using online software I-TASSER and the visualization process was achieved by software DS.

### The Prediction of Allergenicity and Antigenicity

After the construction process of vaccine, the property of vaccine needs to be evaluated for launching later animal experiments smoothly. The allergen of vaccine construct was predicted by online software Al-lerTOP2.0 (http://www.ddg‐pharmfac.net/AllerTOP/). In addition, we used the online software VaxiJen2.0 (http://www.ddgpharmfac.net/vaxijen/VaxiJen/VaxiJen.html) and ANTIGENpro (http://scratch.proteomics.ics.uci.edu/index.html) to predict the antigenicity of vaccine construct.

### The Evaluation of Vaccine Property

#### Animals

In this study, 7 weeks old BALB/C mice were provided by the Animal Experiment Center of Xinjiang Medical University. The mice were randomly divided into two groups. (1) Immunized group (n=5): 20 µg polypeptide vaccine (the polypeptide vaccine was dissolved using normal saline, 0.2 mg/ml) was used for intranasally dripping every 2 weeks for a total of 3 times. (2) Non-immunized group (n=5): The mice did not receive any medication measure, it just was dripped intranasally using sterile water synchronously. The designed polypeptide vaccine was synthesized by SynPeptide Co Ltd (Shanghai, China).

In addition, the mice were immunized with complete and incomplete Freund's adjuvant. In this method, 7 weeks old mice were also randomly divided into immunized group and non-immunized group. (1) Immunized group (n=20): 25 µg vaccine polypeptide with complete Freund's adjuvant (CFA) was subcutaneously injected. The booster immunization of 25 μg vaccine polypeptide with incomplete Freund's adjuvant (IFA) were separately administered 2 and 4 weeks after the primary immunization. (2) Non-immunized group (n=20): the mice were separately injected with CFA and IFA at the same time. After completed last immunization 2 weeks, the blood serum and the splenocytes was harvested from 10 mice in Immunized and 10 mice in Non-immunized group for FCM, ELISA and ELISPOT tests. Another 10 mice in Immunized and 10 mice in Non-immunized were used for challenge with *E. g* protoscolices and establishing mouse infection model.

The whole process of the mouse experiment strictly followed the animal experiment requirements and operating guidelines of the Animal Ethics Committee of First Affiliated Hospital of Xinjiang Medical University. This study was approved by the Animal Ethics Committee of First Affiliated Hospital of Xinjiang Medical University.

#### Patients

The study involved 20 patients with cystic echinococcosis (aged 18-60 years; cystic echinococcosis (CE) group) and 20 healthy people (aged 18-60 years; health control (HC) group). They were from the inpatient and outpatient physical examination population of the First Affiliated Hospital of Xinjiang Medical University from October to December 2020. The selection of CE group strictly followed the clinical diagnosis requirements and these patients excluded other diseases that might affect the experimental results. The peripheral blood of CE group and HC group were collected (4 ml per person). For the ELISA and Western blotting tests, the Ficoll paque was used to separate peripheral blood mononuclear cells (PBMCs) and the blood plasma of each sample was collected at the same time. This study was approved by the Ethics Committee of First Affiliated Hospital of Xinjiang Medical University.

#### Detection of the Specific CD4^+^ and CD8^+^ T-Cell Level With FCM

To verify optimal vaccine immunity composed of both humoral and cellular responses, the Flow cytometry (FCM) was carried out to measure the levels of specific CD4^+^ and CD8^+^ T-cells after vaccination. The splenocytes of mice in Immunized and Non-immunized were co-cultured with the vaccine polypeptide. First, the 96-wells plate was pre-coated with anti-CD3 (OKT3, eBioscience, San Diego, USA, 0.5 μg/mL) monoclonal antibodies (mAbs), and anti-CD28 mAbs (CD28.2, eBioscience, San Diego, USA, 0.5 μg/mL) were added. Then the plate was incubated at 37°C for 72h under 5% CO_2_. The splenocytes suspension were prepared (2×10^6^), and the anti-CD3 (APC), anti-CD4 (FITC) and anti-CD8 (APC-Cy^TM^7) mAbs were added. The splenocytes were incubated 20 min with antibodies, and was centrifuged. After washed by phosphate buffered saline (PBS) 2 times, the splenocytes were re-suspend by 500 μl PBS. With gating of CD3/CD4 and CD3/CD8, the levels of CD3^+^-CD4^+^-T and CD3^+^-CD8^+^-T-cell in immunized and Non-immunized groups were analyzed.

#### Detection of the Antibody Titer With ELISA

In order to verify whether the vaccine polypeptide can stimulate humoral immunity and produce antibodies, the enzyme-linked immuno sorbent assay (ELISA) was conducted to determine the antibody titer. First, the ELISA plate was coated with vaccine polypeptide (5 μg/ml) in coating buffer overnight at 4-8°C. The plate was blocked with phosphate buffer saline (PBS) containing 5% nonfat milk at 37°C for 3h and washed by PBS containing 0.05% Tween 20. Then, after adding the mice blood serum sample (the mice blood serum was diluted in double dilution method), the ELISA plate was incubated at 37°C for 1h and was washed by PBS containing 0.05% Tween 20. The Rabbit anti-Mouse IgG (BD) secondary antibody that labelled with horse radish peroxidase (HRP) was added at a ratio of 1:1000 and was incubated at 37°C for 30 minutes. After washing the ELISA plate again, the tetramethylbenzidine (TMB) solution (sigma) was added and incubated for color development. The OD_450nm_ value was measured using an ELISA plate reader. The antibody titer of IgG was reflected by the maximum dilution ratio of blood serum. Meanwhile, to verify whether the antibodies raised by the vaccine polypeptide were specifically against *E. g.* We conducted ELISA experiment to determine specific anti-*E. g* antibody. As in the previous method, the patient blood plasma sample (the patient blood plasma was diluted in double dilution method) and the Mouse anti-Human IgG (BD) secondary antibody that labelled with HRP were used for experiment. The OD_450nm_ value was also measured using a Microplate reader and to analysis the antibody titer.

#### Detection of the Specific B-Cell Response With ELISPOT

In order to further verify that the vaccine polypeptide can stimulate the body to produce specific anti-*E. g* antibody, the Enzyme-Linked Immunospot (ELISPOT) experiment was carried out. The ELISPOT plate was coated with vaccine polypeptide (5 μg/ml) in coating buffer overnight at 4-8°C. The plate was washed and blocked with 100 μl of RPMI 1640 medium (Sigma Aldrich, St. Louis, UA) containing 10% fetal calf serum (FCS) (Sigma) for 1h at 37°C. The plate was washed by PBS containing 0.05% Tween 20. The mice splenocytes suspension (2×10^5^) and the vaccine polypeptide were added in plates, and every samples were added to three repeated wells. The plate was incubated for 20h at 37°C under 5% CO_2_. The biotinylated detecting antibody (anti-IgG) and Streptavidin-ALP was added. The plate was incubated at 37°C for 30 minutes and the BCIP/NBT (mlbio) solution was added and incubated for color development. Last, antibody secreting cell (ASC) spot were counted using an ELISPOT automatic plate reader (AID Elispot Reader, AID, Germany). The Mouse IgG ELISpot BASIC kit (ALP) (3825-2A, Mabtech, Stockholm, Sweden) was adopted for the ELISPOT experiment.

#### Detection of the IFN-γ With ELISA

In order to verify whether the vaccine polypeptide can stimulate helper T cell immunity, the ELISA experiment was conducted to determine IFN-γ. In 96-well plates with RPMI1640 medium containing 10% FCS, we co-culture the synthesized vaccine polypeptide (5 μg/ml) with PBMCs (2 × 10^5^) of CE and HC groups. After culturing at 37°C for 20h under 5% CO_2_, the ELISA plate was coated with the specific anti-Human IFN-γ capturing antibody in coating buffer overnight at 4-8°C. The plate was blocked with phosphate buffer saline (PBS) containing 5% nonfat milk for at 37°C for 3 h and washed by PBS containing 0.05% Tween 20. Then, the collected cell culture supernatant was added and the plate was incubated at 37°C for 1 h. After washing the ELISA plate with PBS containing 0.05% Tween 20, the specific anti-IFN-γ secondary antibody labelled with HRP was added and incubated at 37°C for 1 h. Last, after washing again and the TMB solution (sigma) was added for color development. The OD_450nm_ value was measured using a Microplate reader and the levels of IFN-γ was reflected by standard curve. The Human IFN-γ ELISA Kit (70-EK180-48, MultiSciences, Hangzhou, China) was adopted for the ELISA experiment.

#### Detection of the Perforin and Granzyme-B With ELISA

In order to verify whether the vaccine polypeptide can stimulate cytotoxic T-cell immunity, we used an ELISA experiment to determine Perforin and Granzyme-B. First, we co-culture the synthesized vaccine polypeptide (5 μg/ml) with PBMCs (2 × 10^5^) of CE and HC groups. After culturing at 37°C for 72 h, the cell culture supernatant was collected. And then the ELISA plate was coated with anti-Human Perforin (clone Pf-80/164, 4 μg/ml) or Granzyme-B capturing antibody (clone GB10, 2 μg/ml) in coating buffer overnight at 4-8 °C. The plate was blocked with phosphate buffer saline (PBS) containing 5% nonfat milk for at 37°C for 3h and washed by PBS containing 0.05% Tween 20. Then the cell culture supernatant was added and the plate was incubated at 37°C for 1h. After washing the ELISA plate with PBS containing 0.05% Tween 20, the secondary antibody labelled with HRP that anti-Perforin (1 μg/ml; clone Pf-344) or anti-Granzyme-B detecting antibody (1 μg/ml; clone GB11) were added and incubated at 37°C for 1 h. After washing again, the TMB solution (sigma) was added for color development. The OD_450nm_ value was determined by Microplate reader and the sample concentration was determined based on the standard curve. The Human Perforin ELISA pro kit (3465-1HP-2, Mabtech, Stockholm, Sweden) and Human Granzyme-B ELISA development kit (3485-1H-6, Mabtech, Stockholm, Sweden) were adopted for the ELISA experiment.

#### Detection of Specific Antibody Response With Western Blotting 

The specific antibody responses against designed recombinant in serum samples of infected patients was assessed using Western blotting. The vaccine polypeptide was separated using SDS-PAGE, and then it was transferred to PVDF membrane (Invitrogen, California, USA). The PVDF membrane was blocked at 37°C for 1 h with 5% skimmed milk powder solution, and it was washed 3 times with TBST for 5 min each time. The PVDF membrane was immersed in human serum (1:400 dilution) of CE group and HC group, incubated at 37°C for 1 h, and washed with TBST 3 times for 5 min each time. The secondary antibody was Rabbit anti-Human and labeled with HRP. The PVDF membrane was incubated with secondary antibody (1:2000 dilution) at room temperature for 1 h, and then was washed by TBST 3 times for 5 min each time. The protein band was visualized with an ECL detection kit (Biosharp, Beijing, China), and exposed on Amersham Hyperfilm (GE Healthcare).

#### Detection of the Weight of Hydatid Cyst

To evaluate potential protective effects of designed vaccine which could induce hosts resistant to a challenge infection with *E. g* protoscolices. All mice of infection model in 2 groups were dissected and the weight of *E. g* cysts was measured after 23 weeks of protoscolices infection.

## Results

### The Amino Acid Sequences of Proteins 

The accession of Protein EgTeg was AY874524 and version was AAX20156.1. The amino acid sequence was ATDPTTMSRSEVEV LKSDMPTEMKNFIIDQVDDTLREYNADSSRPIKLESLVTQLGRTLKQRYEGVWQVVILT GSYSAFSAYTPERLFHFKFGRFVVLVWQSSTY. The accession of Protein EgFABP1 was Q02970 and version was Q02970.2. The amino acid sequence was MEAFLGTWKMEKSEGFDKIMERLGVDFVTRKMGNLVK PNLIVTDLGGGKYKMRSESTFKTTECSF KLGEKFKEVTPDSREVASLITVENG VMKHEQDDKTKVTYIERVVEGNELKATVKVDEVVCVRTYSKVA.

### The Prediction of Physicochemical Parameters of Proteins

Protein EgTeg had 105 amino acids and the total number of atoms of Protein EgTeg is 1705, formula was C_547_H_848_N_142_O_165_S_3_, molecular weight was 12KDa. Theoretical pI was 5.80, including 13 acidic (negatively charged residues) amino acids (Asp + Glu) and 12 alkaline (positively charged residues) amino acids (Arg + Lys). The instability index (II) was computed to be 33.96 (when it < 40, the protein is stable), so this classifies the protein was stable protein. Aliphatic index was 79.71 and Grand average of hydropathicity (GRAVY) was −0.309 (the figure of GRAVY is −2−2, negative value means that protein is hydrophilic), so EgFABP1 belonged to hydrophilic protein.

Protein EgTeg had 133 amino acids and the total number of atoms of Protein EgFABP1 was 2133, formula was C_664_H_1080_N_176_O_205_S_8_, molecular weight was 15KDa. Theoretical pI was 7.70, including 22 acidic (negatively charged residues) amino acids (Asp + Glu) and 23 alkaline (positively charged residues) amino acids (Arg + Lys). The instability index (II) was computed to be 19.89 (when it < 40, the protein is stable), so this classifies the protein was stable protein. Aliphatic index is 75.26 and Grand average of hydropathicity (GRAVY) was −0.427 (the figure of GRAVY is −2−2, negative value means that protein is hydrophilic), so EgFABP1 belonged to hydrophilic protein.

### The Prediction of Transmembrane Domains and Signal Peptide of Proteins

The online server TMHMM showed that both Protein EgTeg and EgFABP1 did not have transmembrane domains. The online server signalP-5.0 showed they also did not have signal peptide (**Supplementary Figures 1, 2**).

### The Prediction of Phosphorylation Sites of Proteins

The analyzed result of online server NetPhos 3.1 showed that Protein EgTeg had 9 Serine and 5 Threonine phosphorylation sites, the main phosphorylation sites of the protein were protein kinase C (PKC) and protein kinase A (PKA). Protein EgFABP1 had 6 Serine, 6 Threonine and 1 Tyrosine phosphorylation sites, the main phosphorylation sites of the protein were protein kinase C (PKC), CDK inhibitor (CKI), insulin receptor (INSR) and protein kinase A (PKA) (**Supplementary Figure 3**).

### The Prediction of Secondary Structure of Proteins

The predicted result of secondary structure of protein by DNASTAR was shown in **Supplementary Figure 4**, it included Gramier-Robson and Chou-Fasman two results. In addition, the online software SOPMA also showed that there were 35.24% Alpha helix, 23.81% Extended strand, 6.67% Beta turn and 34.29% Random coil in Protein EgTeg. And there were 26.32% Alpha helix, 34.59% Extended strand, 9.77% Beta turn and 29.32% Random coil in Protein EgFABP1 (**Supplementary Figures 5-1**, **5-2**). All of the sequences of secondary structure of Protein EgTeg and EgFABP1 were shown in **Supplementary Tables 1-1**, **1-2**.

### The Prediction of Tertiary Structure of Proteins

**Figure 1:**
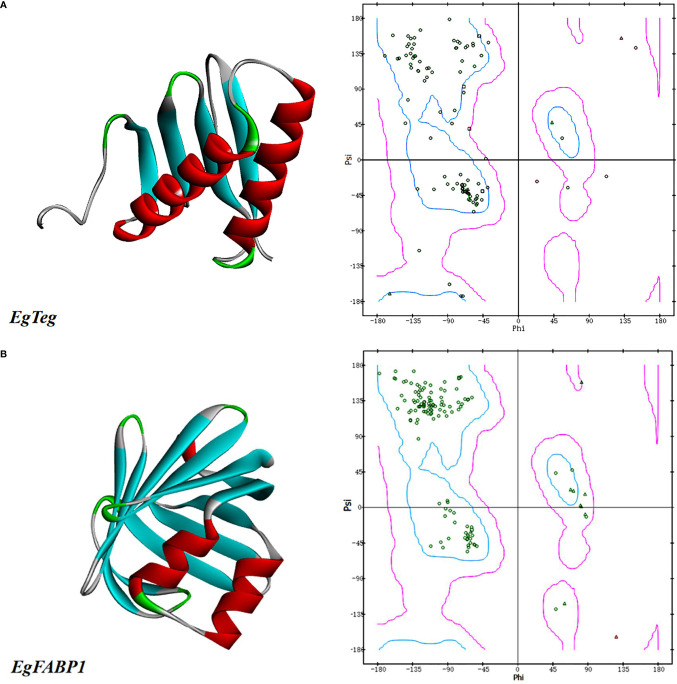
**(A)** The predicted tertiary structures of Protein EgTeg. **(B)** The predicted tertiary structures of Protein EgFABP1. The left part is tertiary structure of protein and the display style is Solid ribbon. The right part is Ramachandran plot, Phi represents the rotation angle of C-N bond on the left side of α-carbon in a peptide unit and Psi represents the rotation angle of C-C bond on the right side of α-carbon. In this Ramachandran plot, the area inside the blue coil is completely allowed, the area inside the purple coil is allowed and the area outside the purple coil is not allowed. When the scatter in the blue coil and the purple coil exceeds 90%, the tertiary structure of the model conforms to the rule of stereochemistry. (For understanding the reference color in remark of the figure clearly, please readers refer to the web version of this article).

The homology-modeling of online software SWISS-MODEL was used to predict the tertiary structure of Protein EgTeg, but the homology of all the protein models found was lower than 30% with Protein EgTeg, so we used the folding-method of online software I-TASSER again to gain the tertiary structure of protein. The database NCBI had already included the tertiary structure of Protein EgFABP1, it had been determined by Jakobsson et al. (18) using the X-raycrystallography. Finally, all the protein molecular models that we obtained were viewed by software DS clearly. And we also plotted Ramachandran plot to evaluate the rationality of structure, the result was shown in **Figures 1A, B**.

### The Prediction of T-Cell Epitopes of Proteins

The MHC restriction must be taken into account when we start to predict the T-cell epitopes, so the CD8^+^ and CD4^+^ T-cell epitopes should be analyzed separately (22). The CD8^+^ and CD4^+^ T-cell epitopes of proteins EgTeg and EgFABP1 were predicted by online software SYFPEITHI and IEDB. For improving the accuracy of the result, we combined the results of two different software as the final T-cell epitopes. The results of CD8^+^ T-cell epitopes of Protein EgTeg and EgFABP1 were shown in **Supplementary Tables 2-1** and **2-2**. The results of CD4^+^ T-cell epitopes of Protein EgTeg and EgFABP1 were shown in **Supplementary Tables 3-1**, **3-2**. The final T-cell epitopes of Protein EgTeg and EgFABP1 were shown in **Table 1**.

**Table 1:** The predicted final antigen epitopes of proteins.

Category	Software	Protein EgTeg		Protein EgFABP1	
		Position	Amino acid sequence	Position	Amino acid sequence
CD8^+^ T-cell	SYFPEITHI, IEDB	7-16	MSRSEVEVLK	25-37	VDFVTRKMGNLVK
		26-35	FIIDQVDDTL	65-72	FKLGEKFK
		51-63	LVTQLGRTLKQRY	84-93	LITVENGVMK
		82-91	YTPERLFHFK		
CD4^+^ T-cell	SYFPEITHI, IEDB	10-26	SEVEVLKSDMPTEMKNF	27-41	FVTRKMGNLVKPNLI
		64-77	EGVWQVVILTGSYS	115-129	KATVKVDEVVCVRTY
		82-96	YTPERLFHFKFGRFV		
B-cell	DNASTAR, IEDB	5-12	TTMSRSEV	9-16	KMEKSEGF
		16-24	KSDMPTEMK	45-59	LGGGKYKMRSESTFK
		32-46	DDTLREYNADSSRPI	70-81	KFKEVTPDSREV
		55-65	LGRTLKQRYEG	93-102	KHEQDDKTKV

### The Prediction of B-Cell Epitopes of Proteins

The B-cell epitopes of proteins EgTeg and EgFABP1 were predicted by software DNASTAR and IEDB. For improving the accuracy of the result, we combined the results of two different software as the final B-cell epitopes. The results predicted by DNASTAR were shown in **Supplementary Figures 6-1**, **6-2**, the results predicted by IEDB were shown in **Supplementary Figures 7-1 (A1-F1)**, **7-2 (A2-F2)** and the results of B-cell epitopes of Protein EgTeg and EgFABP1 were shown in **Supplementary Tables 4-1**, **4-2**. The final B-cell epitopes of Protein EgTeg and EgFABP1 were shown in **Table 1**.

### The Optimization of CD4^+^ T-Cell Epitopes

#### The Crystalline Structure of HLA-DRB1 Molecular

**Figure 2:**
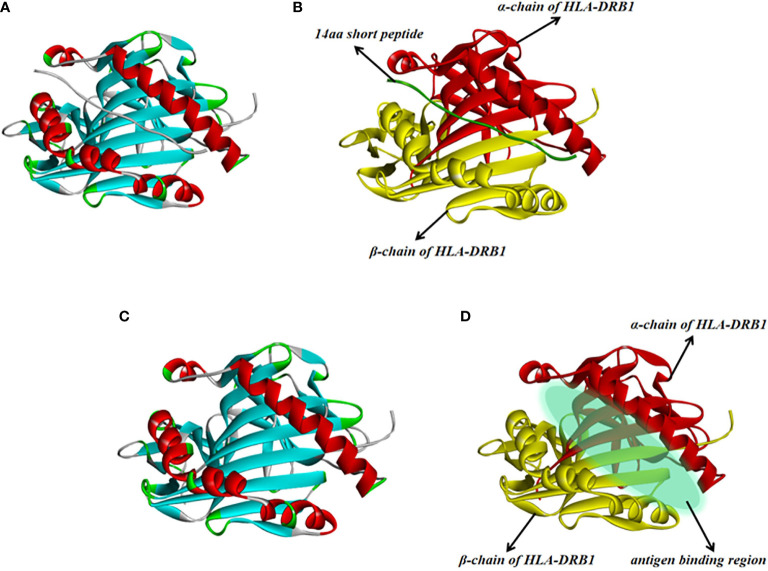
The crystalline structure of HLA-DRB1. The **(A, C)** were general view, the **(B, D)** were specific view in **Figure 2**. According to the figure, the region between the two α-helices is the antigen peptide binding region of HLA-DRB1. (For understanding the reference color in remark of the figure clearly, please readers refer to the web version of this article).

The crystalline structure of HLA-DRB1 molecular that with a peptide was shown in **Figure 2**, the short peptide was HLA Class I histocompatibility antigen A2_104-117_ chain and it had 14 amino acids. The short peptide was knocked out using software DS, the HLA-DRB1 molecular was composed of α-chain and β-chain and the antigen peptide should be bound in the antigen binding region of HLA-DRB1.

#### The Docking Between CD4^+^ T-Cell Epitopes and HLA-DRB1

**Figure 3:**
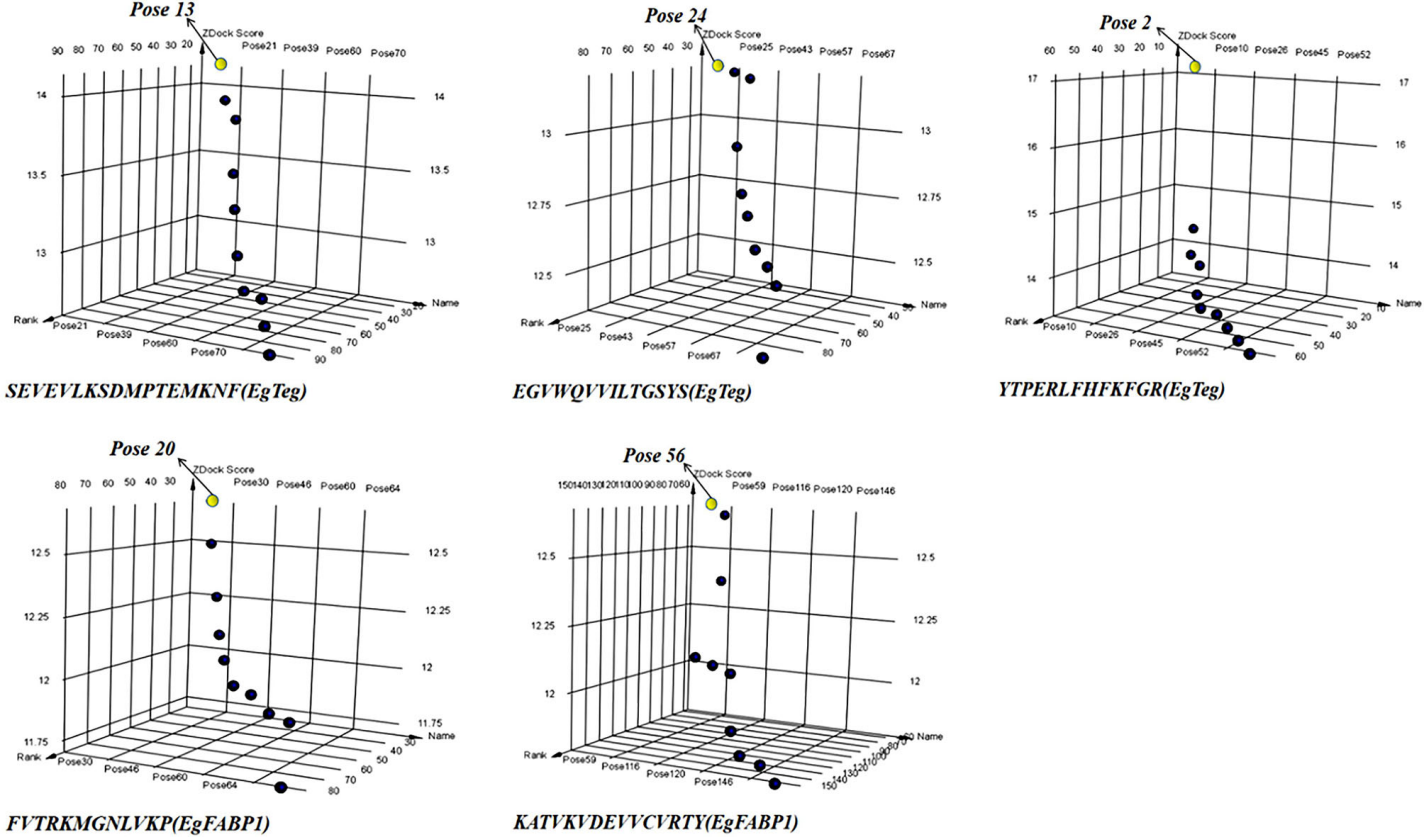
The 3D point plot of docking poses between CD4^+^ T-cell epitopes and HLA-DRB1. All the yellow points were the best Pose in every docking clusters, they are *Pose 13, Pose 24, Pose 2, Pose 20* and *Pose 56* separately. The X-axis is the Pose Name, the Y-axis is the Pose Rank and the Z-axis is the ZDOCK Score. (For understanding the reference color in remark of the figure clearly, please readers refer to the web version of this article).

The molecular docking between preliminary CD4^+^ T-cell epitopes and HLA-DRB1 molecular was carried out by software DS, the CD4^+^ T-cell epitopes that participated in docking included the sequences 10-26, 64-77 and 82-96 of EgTeg, the 27-41 and 115-129 of EgFABP1. The DS generated a series of poses that HLA-DRB1 molecular bound with CD4^+^ T-cell epitopes. We singled out the top ten poses in antigen binding region and made them into a cluster, the best results were *Pose 13, Pose24, Pose 2, Pose 20* and *Pose 56* separately (**Figure 3**). All the poses in this cluster were further optimized by the RDOCK function of DS and the RDOCK results were shown in **Supplementary Table 5**.

#### The Residues of Antigen Peptide Involved in the Interaction

**Figure 4:**
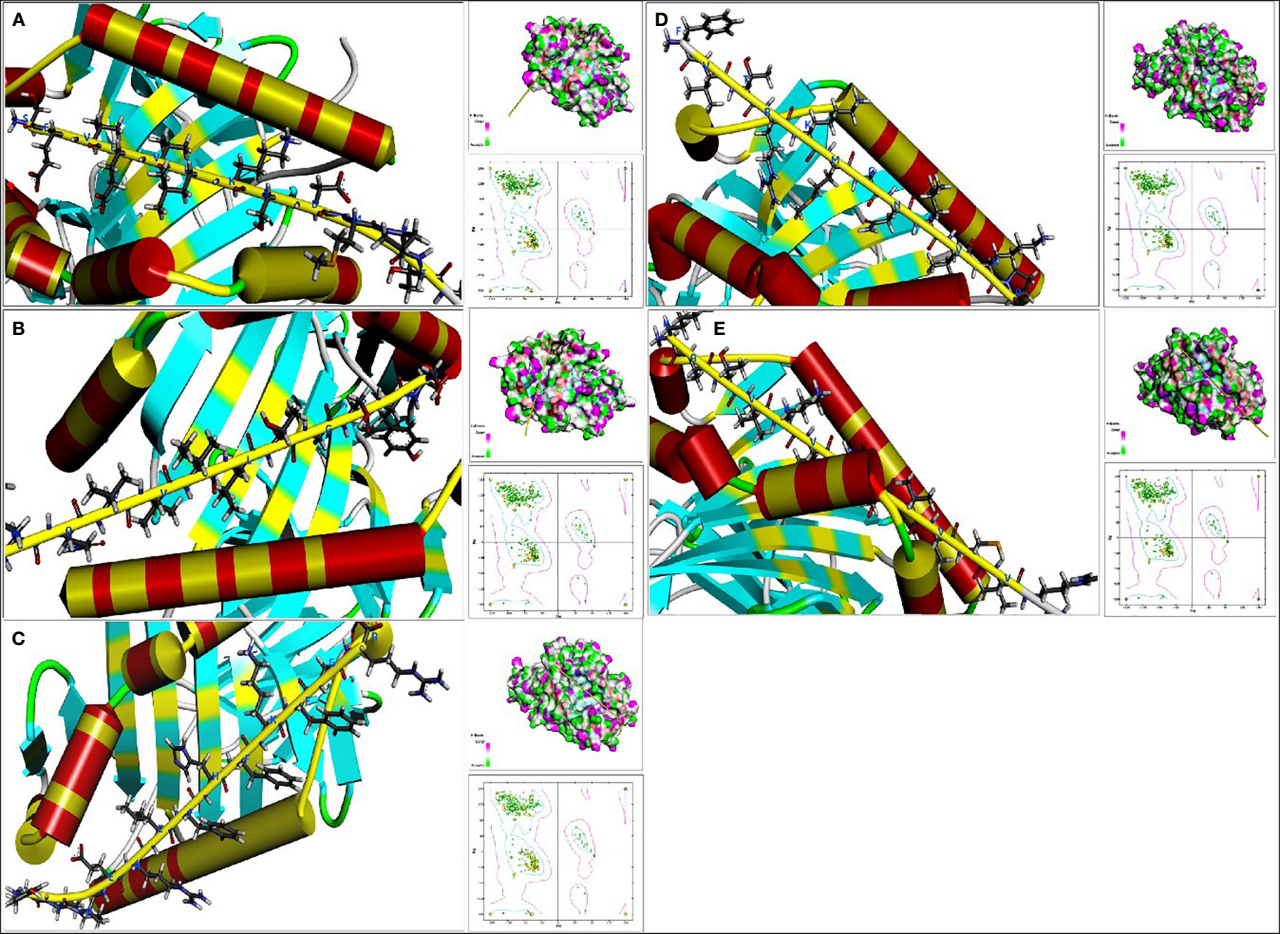
The interaction microstructure between antigen peptide and HLA-DRB1. The **(A)** was *Pose 13*, the **(B)** was *Pose 24*, the **(C)** was *Pose* 2, the **(D)** was *Pose* 20 and the **(E)** was *Pose56.* The yellow chain represents the antigen peptide and the blue abbreviation is the amino acid involved in the interaction. Concurrently there are Hydrogen bond model chart and Ramachandran plot on the right of this Figure. (For understanding the reference color in remark of the figure clearly, please readers refer to the web version of this article).

After applying the RDOCK function of DS to docking poses, we screened some specific amino acid residues of epitopes by calculating the binding site RMSD. These residues participated in the interaction between antigen peptide and HLA-DRB1 molecular, we thought these successive residues had higher possibility to become CD4^+^ T-cell epitopes. These optimized epitopes were the sequences 10-26, 66-77 and 82-94 of EgTeg, the 27-38 and 115-129 of EgFABP1. Through contrastive analysis, we found the new optimized epitopes had highly consistent with the preliminarily screened CD4^+^ TEs, the result was shown in **Table 2**. The interaction microstructure between antigen peptide and HLA-DRB1 were shown in **Figures 4A–E**.

**Table 2:** The contrast of CD4^+^ T-cell epitopes before and after optimization.

Protein	Before optimization	After optimization
EgTeg	10-26	10-26
	64-77	66-77
	82-96	82-94
EgFABP1	27-41	27-38
	115-129	115-129

### The Construction of Multi-Epitope Vaccine

**Figure 5:**
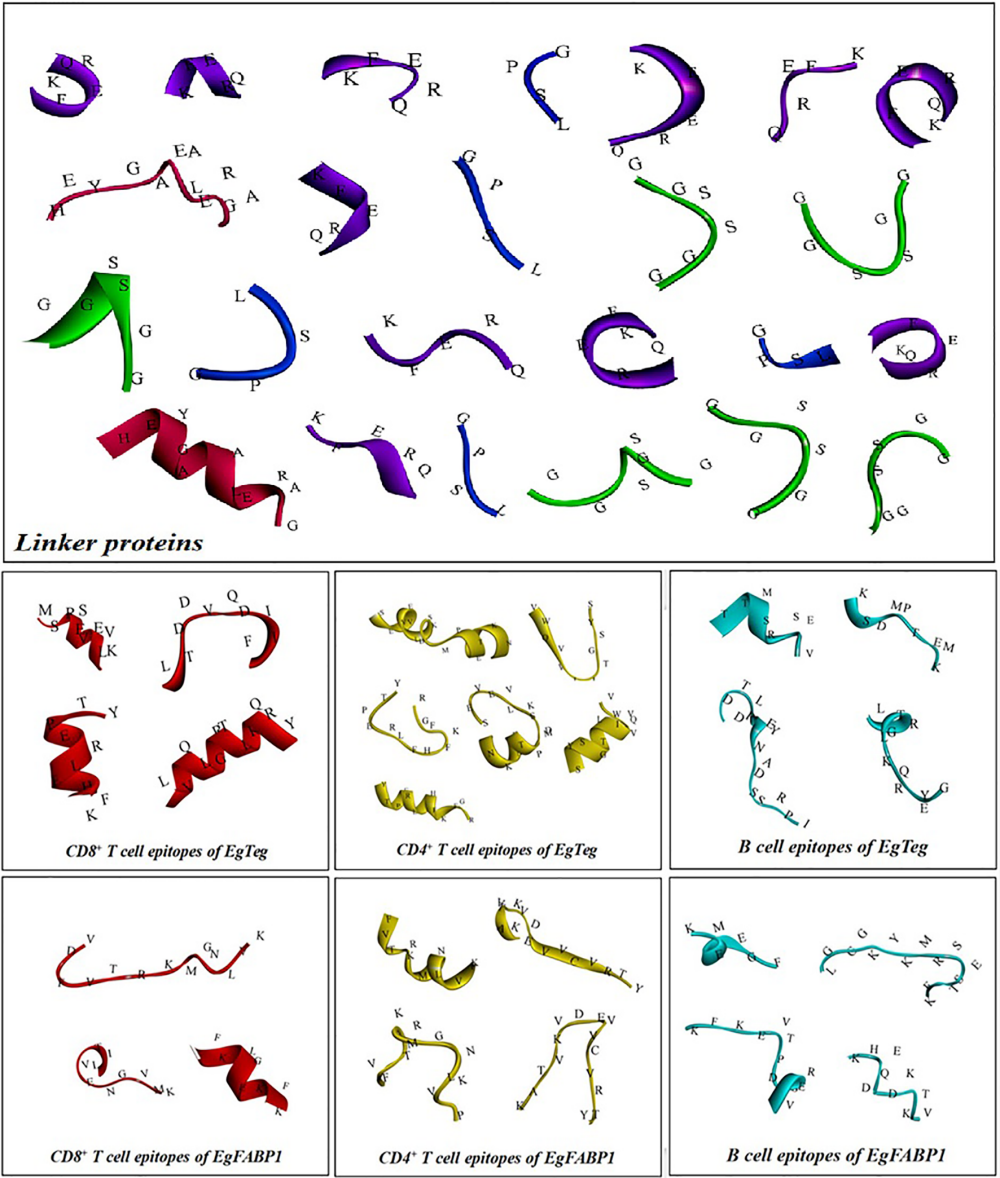
The linker proteins and antigen epitopes in the vaccine.

**Figure 6:**
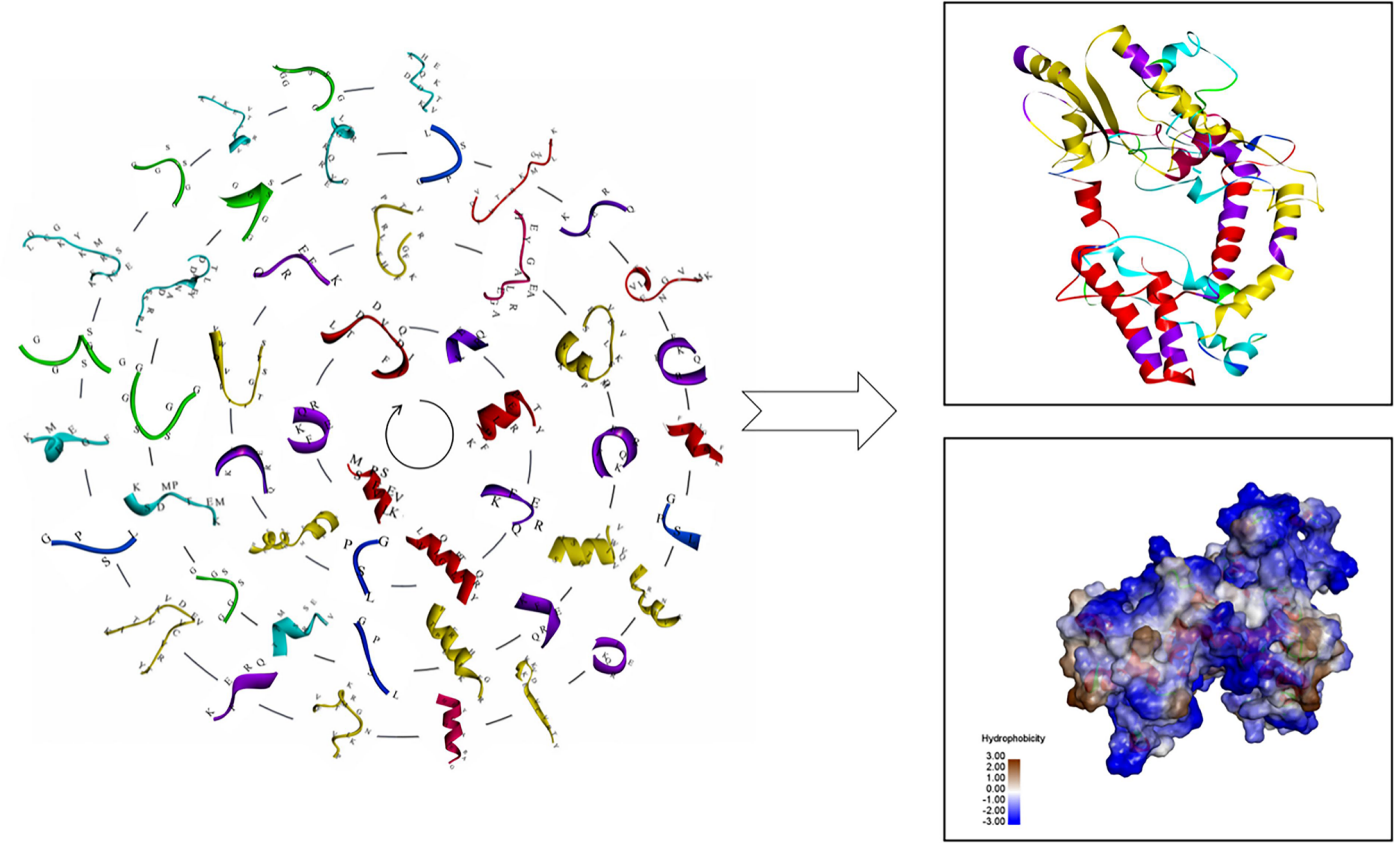
The construction map of the vaccine model. The CD4^+^ TEs, CD8^+^ TEs and BEs of proteins EgTeg and EgFABP1 are linked separately by the specific linker proteins. The linker proteins are *KFERQ*, *HEYGAEALERAG*, *GPSL* and *GGSSGG* and the corresponding amino acid sequences to each antigen peptide were written on the 3D structure of the epitopes. The parts on the right of the figure are the 3D structure of the vaccine, the display style are Flat ribbon and Hydrophobicity chart separately. On the Hydrophobicity chart, the bluer the color, the more hydrophilic it is and the browner the color, the more hydrophobic it is. (For understanding the reference color in remark of the figure clearly, please readers refer to the web version of this article).

The antigen epitopes of proteins EgTeg and EgFABP1 were connected by linker proteins and the compose of the multi-epitope vaccine was CD8^+^ TEs (EgTeg) - CD4^+^ TEs (EgTeg) - CD4^+^ TEs (EgTeg) - BEs (EgTeg) - CD8^+^ TEs (EgFABP1) - CD4^+^ TEs (EgFABP1) - CD4^+^ TEs (EgFABP1) - BEs (EgFABP1). The linker proteins and the related epitopes were shown in **Figure 5**. This vaccine had two repeats of CD4^+^ T cell epitopes of proteins EgTeg and EgFABP1 and it had 435 amino acids and the molecular weight was 49 kDa. The amino acid sequence of this multi-epitope vaccine was written in Supplementary material. The construction map of vaccine was shown in **Figure 6**, including Structure chart and Hydrophobicity chart.

### The Prediction of Allergenicity and Antigenicity

The allergenicity vaccine construct was predicted using online software Al-lerTOP2.0. The Al-lerTOP2.0 defined the vaccine as non-allergen, so the vaccine had high security for human body. The antigenicity of vaccine construct was predicted using online software VaxiJen2.0 and ANTIGENpro. The VaxiJen2.0 showed that overall prediction for the protective antigen was 0.5511 when the threshold value was 0.4 and it belonged to probable antigen. The ANTIGENpro showed that predicted probability of antigenicity was 0.856051 and illustrated that the vaccine had excellent antigenicity. Therefore, the obtained result from the software showed high probability for the antigenic of the vaccine designed.

### The Evaluation of Vaccine Property

#### The Evaluation of Vaccine Induced Humoral Immune Response

**Figure 7:**
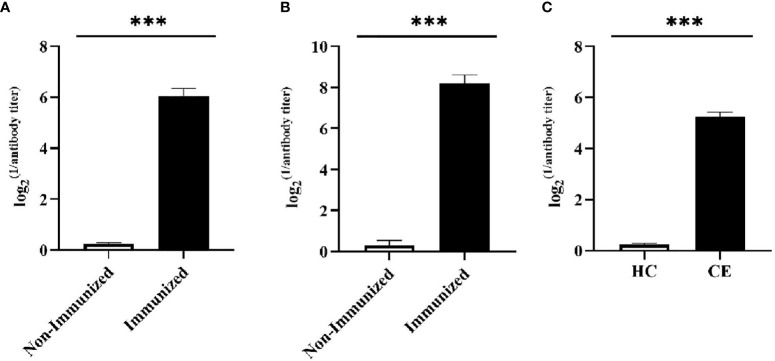
The specific humoral immunity was tested by ELISA. The humoral immunity was assessed by detecting the secretion of specific antibody. **(A)** After the mice were vaccinated by intranasally dripping, the serum antibody level was detected by ELISA. It was found that the serum antibody level of mice in Immunized group was significantly higher than that in Non-immunized group. **(B)** After the mice were vaccinated by subcutaneously injected, the serum antibody level was detected by ELISA. It was found that the serum antibody level of mice in Immunized group was significantly higher than that in Non-immunized group. **(C)** The ELISA was used to detect specific antibody in the blood plasma of CE and HC groups, and it was found that the level of specific antibody in the CE group was significantly higher than that in the HC group. (***P < 0.001).

**Figure 8:**
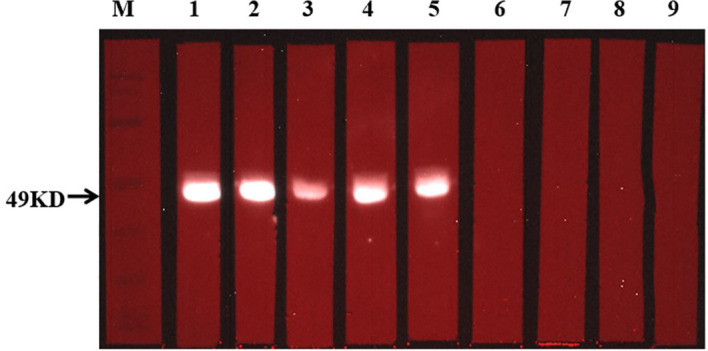
Detection of specific antibody response with Western blotting. The situation of specific antibodies was 49 kDa. In this figure, 1-5 from CE group, and 6-9 from HC group.

**Figure 9:**
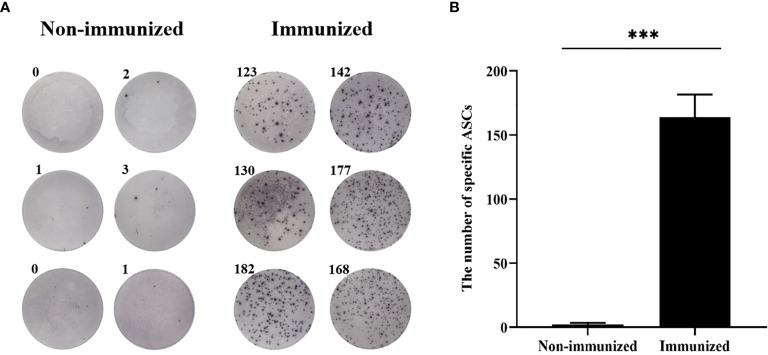
The specific B-cell response was tested by ELISPOT. **(A)** The representative ELISPOT plot of specific B-cell response. **(B)** There were hardly any specific B-cell spots in the Non-immunized group, and the number of ASCs in immunized group was significantly higher than that in Non-immunized group. (***P < 0.001).

Two weeks after the mice completed the immunization, the antibody titer of the mice was detected by ELISA. Meanwhile, the specificity of the vaccine against *E. g* in patient was verified using ELISA and Western blotting. In this study, ELISA test showed that the serum antibody titer of mice in the immunized group was significantly higher than that in the Non-immunized group under the two immunization methods and the difference was statistically significant (*P* < 0.001). In addition, the high level of blood plasma specific anti-*E. g* antibodies was detected in the CE group and it was almost undetectable in the HC group. The difference between CE and HC was statistically significant (*P* < 0.001) (**Figure 7**). Western blotting showed that the specific antibody anti-vaccine antigen was found in the CE group, and there was no specific antibody in HC group (**Figure 8**). The above experiments showed that the vaccine designed in this study could stimulate the body to produce antibodies and the antibodies were specifically against *E. g*. It suggested that the vaccine designed had good antigenicity and immunogenicity. Last, the ELISPOT test showed that the mice immunized with the vaccine could produce specific B-cell response, the difference of specific cell spot count between the Immunized group and the Non-immunized group was statistically significant (*P* < 0.001) (**Figure 9**).

#### The Evaluation of Vaccine Induced CD4^+^ T-Cell Immune Response

**Figure 10:**
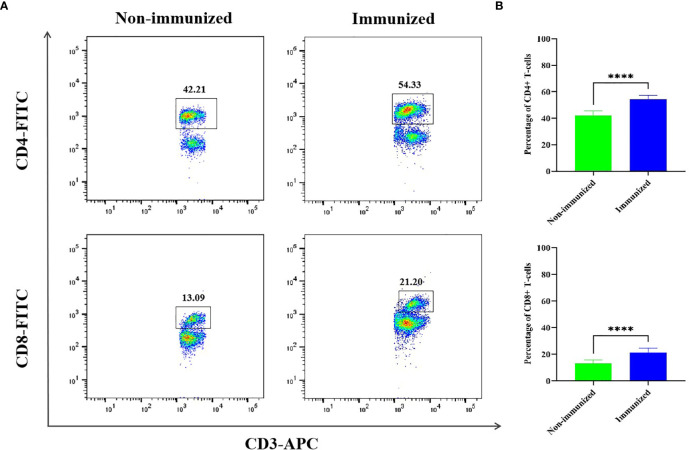
The specific cellular immunity tested by FCM. **(A)** Representative flow cytometric dot-plots of CD3^+^-CD4^+^ and CD3^+^-CD8^+^ T-cells in the mice splenocytes of Immunized and Non-immunized groups were shown. **(B)** The percentage difference of CD3^+^-CD4^+^ and CD3^+^-CD8^+^ T-cells between Immunized and Non-immunized groups was significant. (****P < 0.0001).

**Figure 11:**
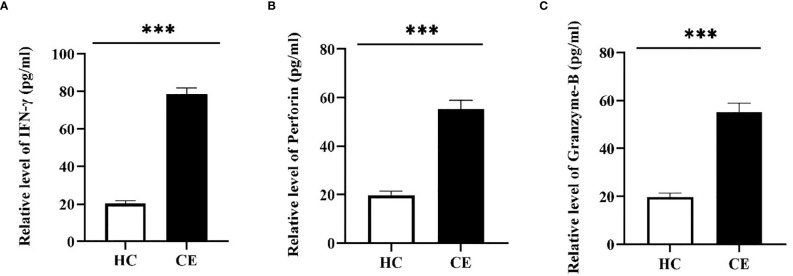
The specific cellular immunity tested by ELISA. The PBMCs of the HC group and the CE group were separated and co-cultured with the vaccine designed in this study. The CD4^+^ T-cell immune response was evaluated by detecting the secretion of IFN-γ in the culture supernatant and the CD8^+^ T-cell immune response was evaluated by detecting the secretion of Perforin and Granzyme-B. **(A)** The relative level of IFN-γ in the HC group and the CE group. **(B)** The relative level of Perforin in the HC group and the CE group. **(C)** The relative level of Granzyme-B in the HC group and the CE group. The relative level of CE group was higher than that of HC group. (***P < 0.001).

The FCM was used to verify the level of specific CD4^+^ T-cells, the result showed that the difference of specific CD4^+^ T-cells level between Immunized group and Non-immunized group was significant (*P* < 0.001) (**Figure 10**). The study evaluated the ability of the vaccine to induce CD4^+^ T-cell immune response and detected the IFN-γ released by patients’ PBMCs through ELISA experiment. The PBMCs of infected patients contain activated CD4^+^ T-cells, so if they come into contact with specific antigens again, they theoretically will release a large amount of IFN-γ. Through co-cultivation of PBMCs and vaccine polypeptide, it was found that the level of IFN-γ in the CE group was significantly higher than that in the HC group using ELISA experiment and the difference was statistically significant (*P* < 0.001). The result was showed in **Figure 11A**. It suggested that the vaccine designed in this study can stimulate CD4^+^ T-cell to release IFN-γ and cause cellular immune responses and has good immunogenicity. The result indicated that the vaccine designed in this study was effective in stimulating CD4^+^ T-cell to produce an immune response.

#### The Evaluation of Vaccine Induced CD8^+^ T-Cell Immune Response

The FCM was used to verify the level of specific CD8^+^ T-cells, the result showed that the difference of specific CD8^+^ T-cells level between Immunized group and Non-immunized group was significant (*P* < 0.001) (**Figure 10**). In this study, the PBMCs from CE and HC groups were co-cultured with the designed vaccine polypeptide and the relative content of Perforin and Granzyme-B in the culture supernatant was detected by ELISA experiment to evaluate the immune response of CD8^+^ T-cell. When activated CD8^+^ T-cells contact specific antigens, they will exert their cytotoxic effects by releasing perforin and granzyme. We tested the content of Perforin and Granzyme-B in the supernatant of the CE group and the HC group and found that the relative level of Perforin and Granzyme-B in the CE group were significantly higher than those in the HC group and the difference is statistically significant (*P* < 0.001). The result was showed in **Figures 11B, C**. These results indicated that the vaccine designed in this study was effective in stimulating CD8^+^ T-cell to produce an immune response and had good immunogenicity.

#### Evaluation of Potential Protective Effects of Designed Vaccine

**Figure 12:**
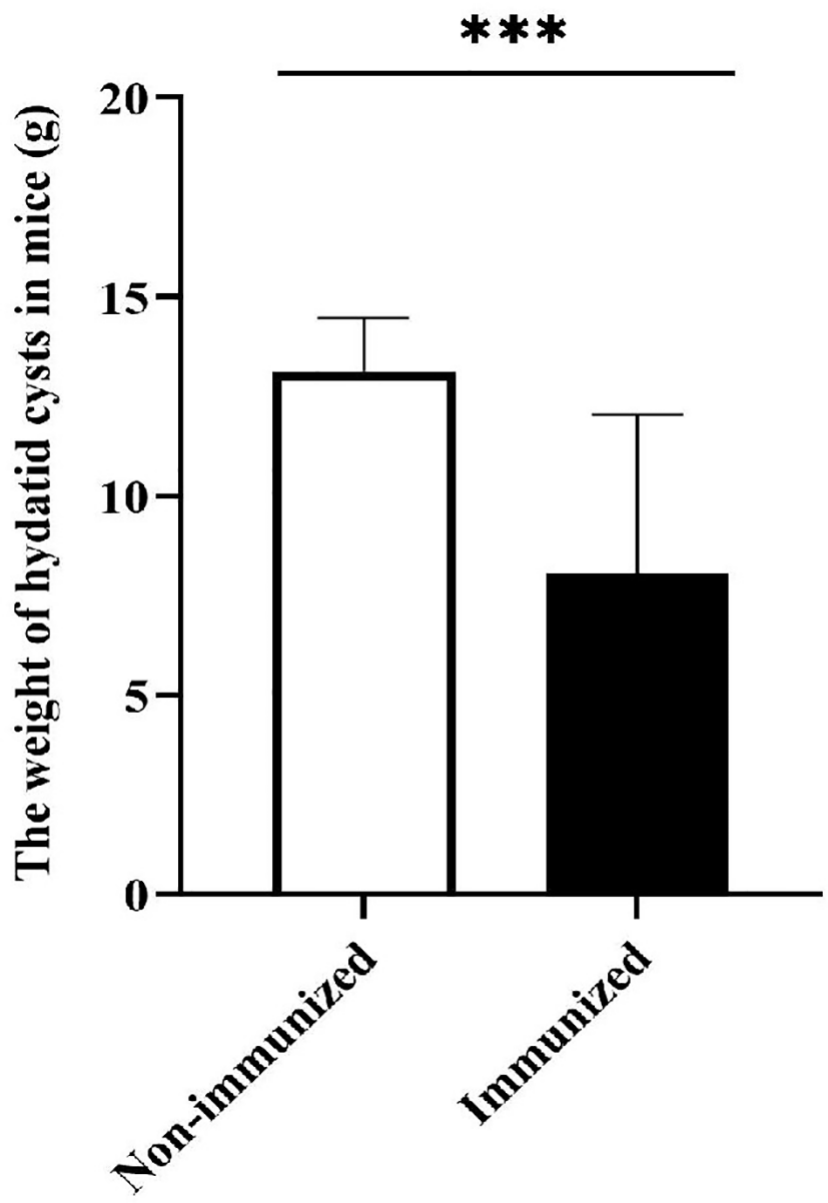
The weight of hydatid cysts. The difference in the weight of hydatid cysts between Immunized group and Non-immunized group was statistically significant. (***P < 0.001).

In order to verify the potential protective effects of the vaccine designed in this study, we established an animal infection model of mice challenged with *E. g* protoscolices. The results showed that there was a significant difference in the weight of hydatid cysts between Immunized group and Non-immunized group (*P* < 0.001), the result was showed in **Figure 12**. The average hydatid cyst weight of immunized mice was less than that of non-immunized mice, which indicated that the vaccine had good protective effect and could prevent the infection of *E. g* protoscolices properly.

## Discussion

Hydatidosis predominates in nearly all over the globe. Control of the hydatidosis, especially in developing countries, necessitates developing of special effective tools for protection of communities from hydatid disease. Vaccine is still the first choice for echinococcosis prevention. In this study, for the first time, we predicted the T- and B-cell epitopes of EgTeg, EgFABP1and constructed multi-epitope vaccine. The experiments *in vitro* and *vivo* were carried out proved that the multi-epitope vaccine had good antigenicity and immunogenicity. In recent years, with the development of molecular immunology technology in leaps and bounds, immunology methods are becoming more and more important in the diagnosis and treatment of hydatid disease. Using the techniques and methods of immunoinformatics to design novel multi-epitope vaccine that composed of several single proteins has become a new approach to elicit a protective immune response (23, 24). It had been proved that Protein EgTeg was involved in the early inflammatory response in the process of hydatid infection and achieves immune escape through a variety of immune regulation and triggers and maintains Th2 cells’ polarization (25). E. g’s hydatid cysts had high requirements for its parasitic environment, which requires Lipid Transfer Protein (LTP) to transport lipids in keeping biosynthetic processes. EgFABP1 was a kind of LTP that expressed at the tegumental level in the protoscolex (26) and it was a promising candidate protein for hydatid vaccine (18). So, our study was committed to use immunoinformatics methods to predict epitopes and design an anti*-E. g* multi-epitope vaccine based on Protein EgTeg and EgFABP1.

The online software ProtParam showed that EgTeg and EgFABP1 were stable and hydrophilic protein. The server NetPhos3.1 showed the main phosphorylation sites of Protein EgTeg were protein kinase C (PKC) and protein kinase A (PKA), the main phosphorylation sites of Protein EgFABP1 were protein kinase C (PKC), CDK inhibitor (CKI), insulin receptor (INSR) and protein kinase A (PKA). All of these biochemical parameters will be conducive to the Separation and purification of protein expressed in future research. The online server TMHMM showed that neither Protein EgTeg nor EgFABP1 had transmembrane domains, which may be helpful to the full contact of antigen presenting cells, effector T-cells and B-cells (19), elicit a strong immune response. The results analyzed by server signalP-5.0 showed that there were no signal peptides in Protein EgTeg and EgFABP1, which not interfere with the software prediction function, so we used the whole amino acid sequence in prediction of epitopes.

The secondary structure of EgTeg and EgFABPA1were predicted by DNASTAR and SOPMA. The Protein program of DNASTAR has many different methods to predict secondary structure, such as the Garnier-Robson that calculating the proportion of specific amino acids and the Chou-Fasman that distinguishing the crystal structure of amino acids are common methods to achieve prediction works. In all secondary structures of protein, the stability of Alpha helix and Extended strand are maintained by the hydrogen bonds and mainly locate in the internal space structure of protein. Contrarily, the flexible structure is composed of Beta turn and Random coil that mainly located in the external regions, it is easier to be identified and combined by the leukomonocytes and antibody (27). The SOPMA web server result showed that there are 40.96% flexible structures in Protein EgTeg and 39.09% flexible structures in Protein EgFABP1. The results of software DNASTAR were similar to SOPMA, which probably suggested that there were many of antigen epitopes in these two proteins. Meanwhile, the tertiary structure of two proteins also showed that there were many Beta turn and Random coil structures, which indicated that the two proteins had efficient antigenic potential too.

In the infection process of echinococcosis, T-cell involved in cellular immune has a major function for resistance to infection (28). Due to the existence of multiple alleles in the HLA-complex, the HLA-complex has become the most complex gene system in human with a high degree of polymorphism. And human HLA-I and HLA-II, the distribution frequency of HLA alleles varies with ethnic groups and regions (29). Therefore, we used the online software SYFPEITHI and IEDB to predict the HLA class I restricted HLA-A*1101, HLA-A*0201 and HLA-A*0301 CD8^+^ T-cell epitopes, HLA class II restricted HLA-DRB1*0701, HLA-DRB1*1501 and HLA-DRB1*0301 CD4^+^ T-cell epitopes. Meanwhile, we performed a cross-checking procedure to predict and screen the potent B-cell epitopes using IEDB and DNASTAR servers. The currently recognized four antigen-related parameters Accessibility, Antigenicity, Flexibility and Hydrophilicity are used to analyse B-cell epitopes, which has good predictive effect (30), so the B-cell epitopes were predicted by the four parameters of software IEDB and DNASTAR.

At present, it is believed that the immunogenicity of single epitope is weak, while the combination of T- and B-cell polypeptide epitopes can induce strong humoral and cellular immune responses at the same time (31). Epitopes combined T- and B-cell often have more than 50% immunogenicity of the complete antigen molecule (32–34), so, it has become a hot spot of current vaccine research. Our research combined the results of different predicting tools as the final CD8^+^ T-cell, CD4^+^ T-cell and B-cell epitopes of Protein EgTeg and EgFABP1. According to the research of Pourseif et al. (21), we thought that CD4^+^ T-cell had excellent helpful function in humoral immunity and cellular immunity. Thus, our research not only further optimized the CD4^+^ TEs using software DS, but also applied two repeats of optimized CD4^+^ TEs in the construction of our multi-epitope vaccine. The vaccine included 7-16, 26-35, 51-63 and 82-91 four CD8^+^ TEs, 10-26, 66-77 and 82-94 three CD4^+^ TEs and 5-12, 16-24, 32-46 and 55-65 four BEs of Protein EgTeg. 25-37, 65-72 and 84-93 three CD8^+^ TEs, 27-38 and 115-129 two CD4^+^ TEs and 9-16, 45-59, 70-81 and 93-102 four BEs of Protein EgFABP1.

Thanks to the immune response induced by different antigens can obtain better immune protection effect than single protein antigen, we took the epitopes of proteins EgTeg and EgFABP1 in tandem array for getting stronger immune effect. We evaluated the performance of the vaccine designed in this study through *in vivo* and *in vitro* experiments. In ELISA test, it was found that humoral immunity can be stimulated to produce high level of antibodies by vaccinating mice, and it is special anti-*E. g.* In addition, it was found that the serum antibodies of infected patients can specifically bind to the vaccine and the PBMCs of patients co-cultured with the vaccine can produce high level of IFN-γ, Perforin and Germany-B. In ELISPOT test, the specific B-cell response was evaluated. The FCM test showed that there was significant difference in the levels of specific CD4^+^ and CD8^+^ T-cells between the Immunized group and the Non-immunized group. The Western blotting showed that there were specific antibodies anti-vaccine antigen in CE group. Last, the difference of the hydatid cysts weight showed the potential protective effects in *E. g* infection. The above experiments showed that the vaccine designed in this study can activate the body’s humoral immunity and cellular immunity and has good antigenicity and immunogenicity. At the same time, we predicted the allergenicity of the vaccine and found that the vaccine designed in this study is not an allergen to humans, it suggested that the vaccine had a high biosafety. In short, this study found some dominant epitopes of proteins EgTeg and EgFABP1 by utilizing many of different immunoinformatics tools. The study provided a new thinking for screening of E. g antigen epitopes, laid a foundation for further construction of anti-*E. g* multi-epitope vaccine and accelerates the development of hydatid disease vaccines (35).

## Date Availability Statement 

The raw data supporting the conclusion of this article will be made available by the authors, without undue reservation.

## Ethics Statement

The studies involving human participants were reviewed and approved by Ethics Committee of First Affiliated Hospital of Xinjiang Medical University. Written informed consent for participation was not required for this study in accordance with the national legislation and the institutional requirements. The animal study was reviewed and approved by Animal Ethics Committee of First Affiliated Hospital of Xinjiang Medical University.

## Author Contributions

MY, YZ, YL, ZC, TS, and ZL performed the experiments. MY, YZ, and YL wrote the paper. FZ and JD edited the final version. All authors contributed to the article and approved the submitted version.

## Funding

This work has been supported by the National Natural Science Foundation of China [Grant number 81960373, 81660343, 81460307, 31560262] and the fund of the Xinjiang Key construction Project of the 13th Five-Year Plan (basic medicine).

## Conflict of Interest

The authors declare that the research was conducted in the absence of any commercial or financial relationships that could be construed as a potential conflict of interest.

## Publisher’s Note

All claims expressed in this article are solely those of the authors and do not necessarily represent those of their affiliated organizations, or those of the publisher, the editors and the reviewers. Any product that may be evaluated in this article, or claim that may be made by its manufacturer, is not guaranteed or endorsed by the publisher.
