# Supplementary material for: Design of a Novel Multi-Epitope Vaccine Against Echinococcus granulosus in Immunoinformatics
Source: Front Immunol. 2021 Aug 12;12:668492. doi: 10.3389/fimmu.2021.668492 (PMC8388843; doi:10.3389/fimmu.2021.668492)
Supplement: Supplementary file 1 [file DataSheet_1.pdf]

Supplementary Material

3 Results

3.3 The Prediction of transmembrane domains and signal peptide of proteins

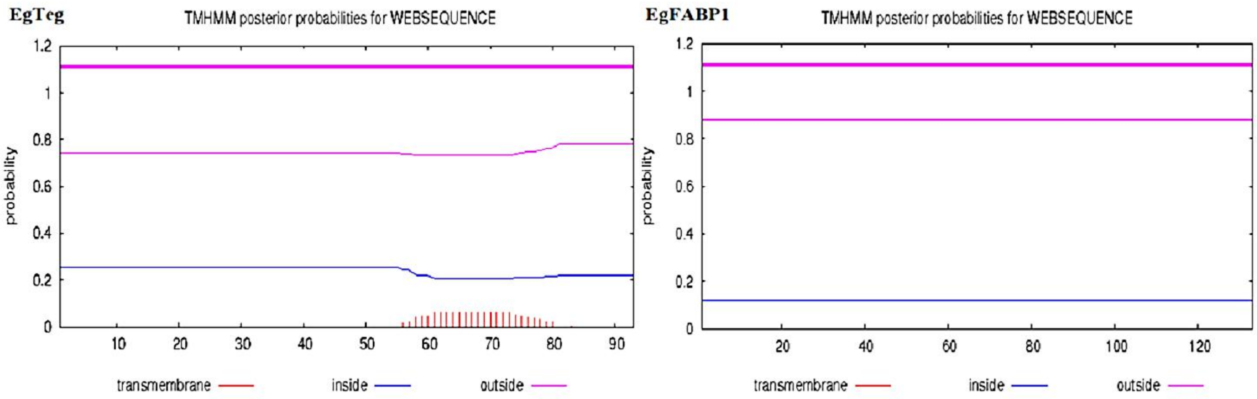

Supplementary Figure 1. The predicted transmembrane domains of Protein EgTeg and EgFABP1.

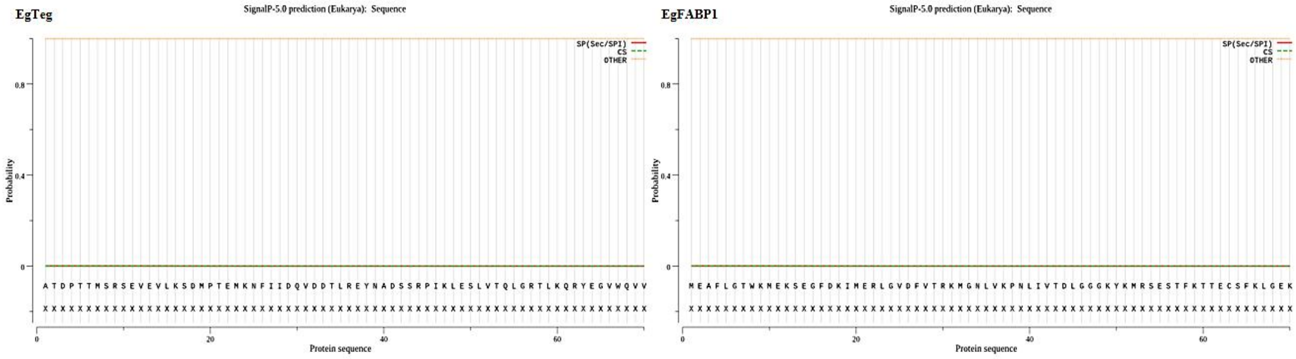

Supplementary Figure 2. The predicted signal peptide of Protein EgTeg and EgFABP1.

3.4 The prediction of phosphorylation sites of proteins

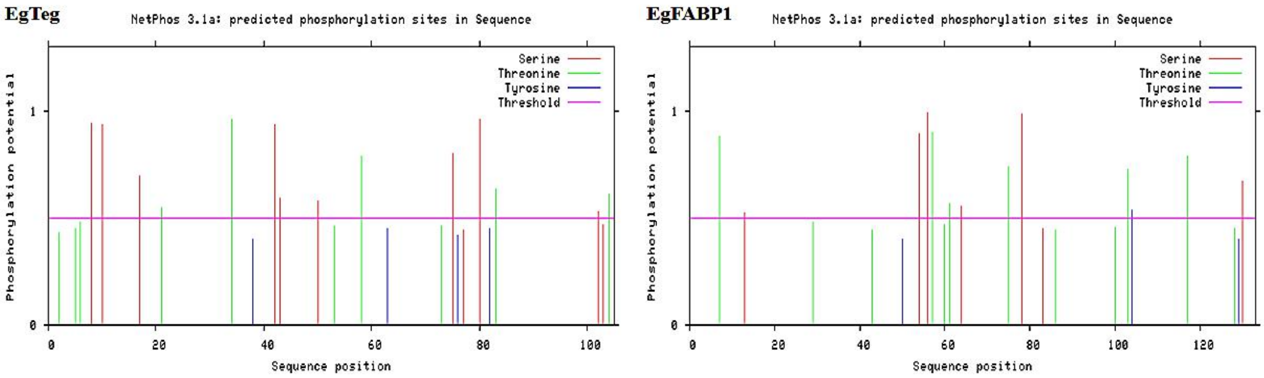

Supplementary Figure 3. The predicted phosphorylation sites of Protein EgTeg and EgFABP1.

3.5 The prediction of secondary structure of proteins

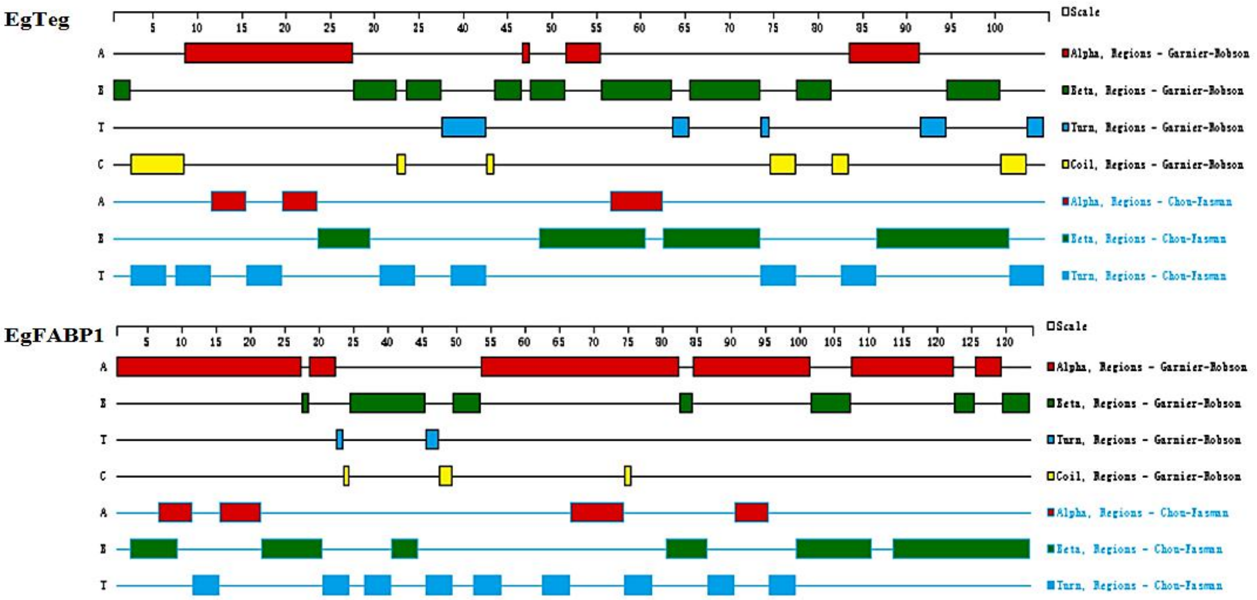

Supplementary Figure 4. The predicted secondary structure of Protein EgTeg by DNASTAR. The DNASTAR was used for the prediction of the secondary structure of Protein EgTeg and EgFABP1. Lines 1, 2, 3, and 4 are the Gramier-Robson method, the red represents the α-helix, the green represents the β-fold, the blue represents the β-turn and the yellow represents the random coil; Lines 5, 6 and 7 are the Chou-Fasman method, the red represents the α-helix, the green represents the β-fold, the blue represents the β-turn and without random coil structure.

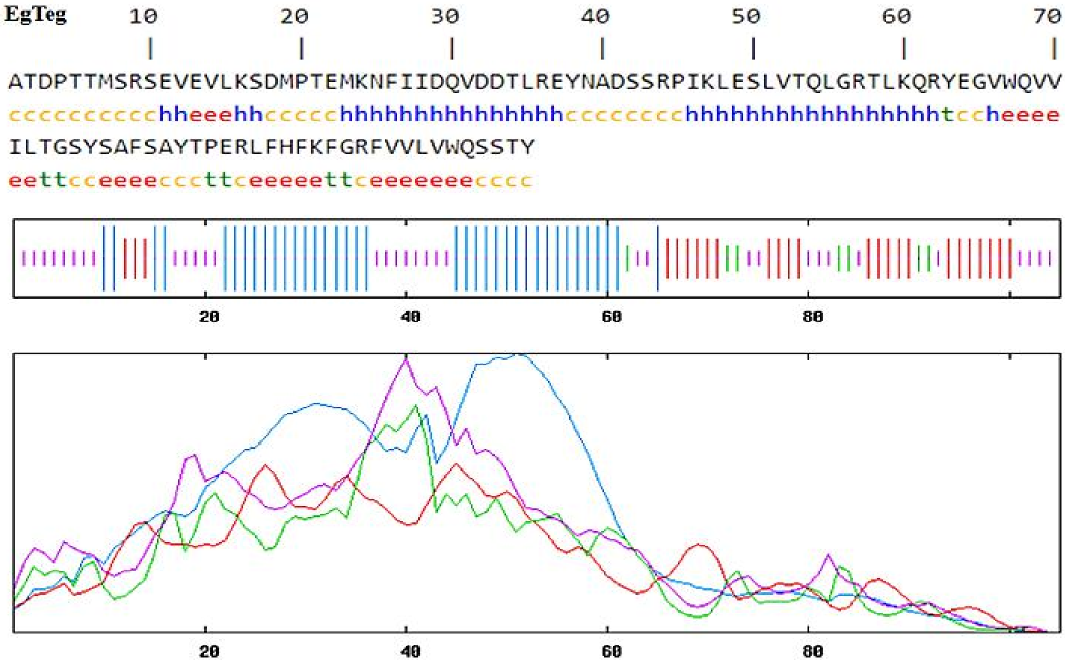

Supplementary Figure 5-1. The predicted secondary structure of Protein EgTeg by SOPMA. The result of server SOPMA indicates that the blue h is Alpha helix and accounts for 35.24% approximately, the red e is Extended strand and accounts for 23.81% approximately, the green t is Beta turn and accounts for 6.67 % approximately, the yellow c is Random coil and accounted for 34.29% approximately.

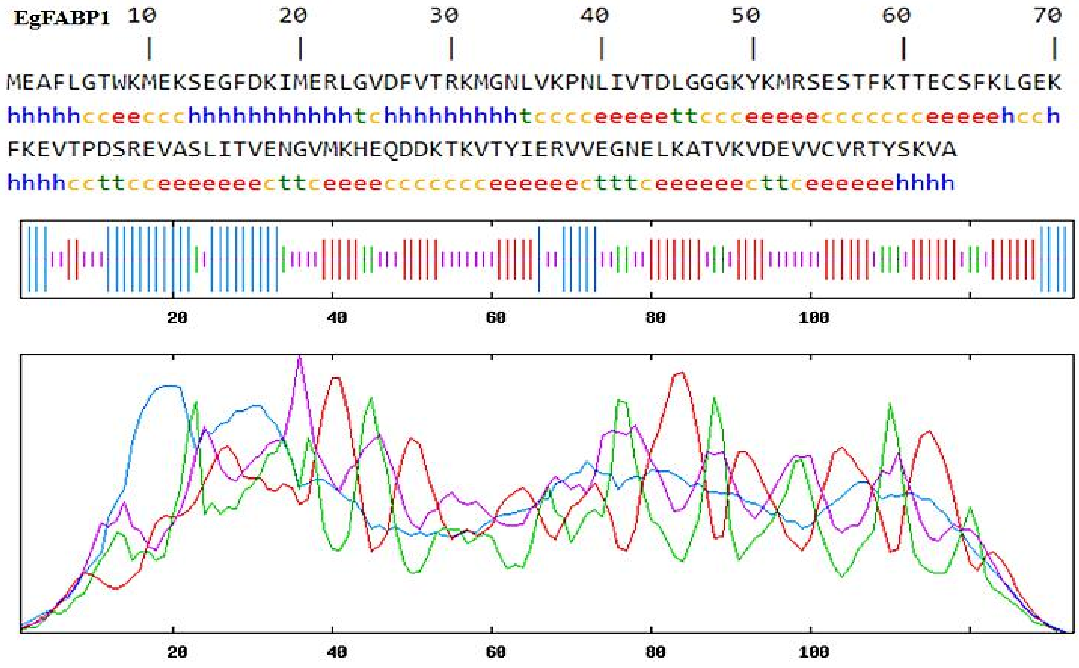

Supplementary Figure 5-2. The predicted secondary structure of Protein EgFABP1 by SOPMA. The result of server SOPMA indicates that the blue h is Alpha helix and accounts for 26.32 % approximately, the red e is Extended strand and accounts for 34.59% approximately, the green t is Beta turn and accounts for 9.77 % approximately, the yellow c is Random coil and accounts for 29.32% approximately.

Supplementary Table 1-1. DNASTAR and SOMPA predicted the secondary structures of Protein EgTeg.

Methods	α-Helix		β-Strand		β-Turn		Random Coil	
DNASTAR								
Garnier-Robson	9-27	52-55	28-31	35-37	38-42	56-57	1-8	32-34
	84-90		48-51	67-72	62-66	72-74	43-45	58-61
			95-99		91-94	104-105	75-78	80-82
							101-102	
Chou-Fasman	11-16	20-23	24-30	49-61	2-7	8-12		
	57-63		63-74	87-101	16-20	31-35		
					39-43	74-78		
SOMPA	10-11	15-16	12-14	66-72	73-74	84-85	1-9	17-21
	22-36	45-61	77-80	87-91	92-93		37-44	63-64
			95-101				75-76	81-83
							92-93	

Supplementary Table 1-2. DNASTAR and SOMPA predicted the secondary structures of Protein EgFABP1.

Methods	α-Helix		β-Strand		β-Turn		Random Coil	
DNASTAR								
Garnier-Robson	1-27	29-33	28-29	35-46	33-34	46-48	48-50	75-76
	54-82	84-102	49-54	83-85				
	108-122	126-130	102-107	123-126				
			130-133					
Chou-Fasman	7-11	16-22	3-10	22-31	12-16	31-34		
	67-74	91-96	41-45	81-87	37-41	46-50		
			100-111	114-133	53-57	63-67		
					75-79	87-91		
					96-99			
SOMPA	1-5	13-23	8-9	40-44	45-46	77-78	6-7	10-12
	26-34	70-74	50-54	62-66	89-90	110-112	36-39	47-49
	130-133		81-87	92-95	121-122		55-61	68-69
			103-108	114-119			75-76	79-80
			124-129				96-102	

3.7 The prediction of T-cell epitopes of proteins

Supplementary Table 2-1. The predicted $CD8^+$ T-cell epitopes of Protein EgTeg by SYFPEITHI and IEDB.

Allele	SYFPEITHI			IEDB		
	Position	Sequence	Score	Position	Sequence	Percentile rank
HLA-A*1101	7-16	MSRSEVEVLK	27	7-16	MSRSEVEVLK	0.61
	51-60	LVTQLGRTLK	22	82-91	YTPERLFHFK	0.67
	82-91	YTPERLFHFK	21	77-86	SAFSAYTPER	0.86
HLA-A*0201	26-35	FIIDQVDDTL	24	60-69	KQRYEGVWQV	0.03
	50-59	SLVTQLGRTL	24	26-35	FIIDQVDDTL	0.62
	6-15	TMSRSEVEVL	22	14-32	VLKSDMPTEM	1.20
HLA-A*0301	96-105	VVLVWQSSTY	25	7-16	MSRSEVEVLK	0.86
	51-60	LVTQLGRTLK	22	51-60	LVTQLGRTLK	0.99
	54-63	QLGRTLKQRY	21	80-89	SAYTPERLFH	1.50

Supplementary Table 2-2. The predicted $CD8^+$ T cell epitopes of Protein EgFABP1 by SYFPEITHI and IEDB.

Allele	SYFPEITHI			IEDB		
	Position	Sequence	Score	Position	Sequence	Percentile rank
HLA-A*1101	28-37	VTRKMGNLVK	25	42-51	VTDLGGGKYK	0.30
	90-99	GVMKHEQDDK	23	22-31	RLGVDFVTRK	0.33
	63-72	CSFKLGEKFK	21	84-93	LITVENGVMK	0.82
HLA-A*0201	19-28	IMERLGVDFV	23	65-74	FKLGEKFKEV	0.73
	31-40	KMGNLVKPNL	20	82-91	ASLITVENGV	1.40
	114-123	LKATVKVDEV	20	31-40	KMGNLVKPNL	1.90
HLA-A*0301	84-93	LITVENGVMK	26	22-31	RLGVDFVTRK	0.03
	22-31	RLGVDFVTRK	25	3-12	AFLGTWKMEK	0.49
	18-27	KIMERLGVDF	13	50-59	YKMRSESTFK	0.53

Supplementary Table 3-1. The predicted $CD4^+$ T-cell epitopes of Protein EgTeg by SYFPEITHI and IEDB.

Allele	SYFPEITHI			IEDB		
	Position	Sequence	Score	Position	Sequence	Percentile rank
HLA-DRB1*0701	67-81	WQVVILTGSYSAFSA	28	85-99	ERLFHFKFGRFVVLV	5.50
	35-49	LREYNADSSRPIKLE	26	87-101	LFHFKFGRFVVLVWQ	5.50
	4-18	PTTMSRSEVEVLKSD	22	84-98	PERLFHFKFGRFVVL	5.50
HLA-DRB1*1501	32-46	DDTLREYNADSSRPI	28	67-81	WQVVILTGSYSAFSA	2.80
	76-90	YSAFSAYTPERLFHF	26	66-80	VWQVVILTGSYSAFS	3.60
	20-34	PTEMKNFIIDQVDDT	24	64-78	EGVWQVVILTGSYSA	4.10
HLA-DRB1*0301	52-66	VTQLGRTLKQRYEGV	26	11-25	EVEVLKSDMPTEMKN	1.80
	12-26	VEVLKSDMPTEMKNF	22	13-27	EVLKSDMPTEMKNFI	1.80
	68-82	QVVILTGSYSAFSAY	22	10-24	SEVEVLKSDMPTEMK	1.80

Supplementary Table 3-2. The predicted $CD4^+$ T-cell epitopes of Protein EgFABP1 by SYFPEITHI and IEDB.

Allele	SYFPEITHI			IEDB		
	Position	Sequence	Score	Position	Sequence	Percentile rank
HLA-DRB1*0701	5-19	LGTWKMEKSEGFDKI	24	28-42	VTRKMGNLVKPNLIV	8.30
	13-27	SEGFDKIMERLGVDF	22	27-41	FVTRKMGNLVKPNLI	11.00
	47-61	GGKYKMRSESTFKTT	22	29-43	TRKMGNLVKPNLIVT	11.00
HLA-DRB1*1501	2-16	EAFLGTWKMEKSEGFTWKM	24	31-45	KMGNLVKPNLIVTDL	15.00
	7-21	EKSEGFDKIMEKYKMRSESTF	24	30-44	RKMGNLVKPNLIVTD	15.00
	49-63	KTTEC	24	29-43	TRKMGNLVKPNLIVT	15.00
HLA-DRB1*0301	71-85	FKEVTPDSREVASLI	29	113-127	ELKATVKVDEVVCVR	0.91
	29-34	TRKMGNLVKPNLIVT	25	115-129	KATVKVDEVVCVRTY	0.91
	115-129	KATVKVDEVVCVRTY	23	114-128	LKATVKVDEVVCVRT	0.91

3.8 The prediction of B-cell epitopes of proteins

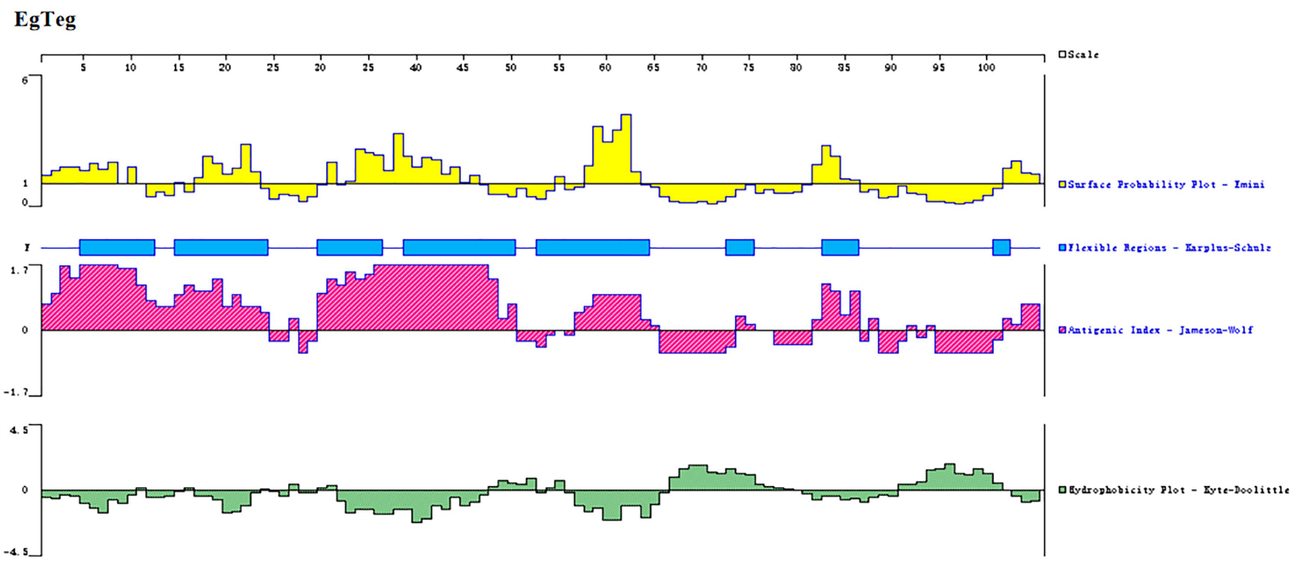

Supplementary Figure 6-1. The predicted B-cell epitopes of Protein EgTeg by DNASTAR. The predicted B-cell epitopes of Protein EgTeg by DNASTAR. The yellow area represents Surface accessibility, the blue area represents Flexibility, the purple area represents Antigenicity and the green area represents Hydrophobicity. We need to obtain the regions above the threshold of the yellow area, the blue area and the purple area, and the regions below the threshold of the green area.

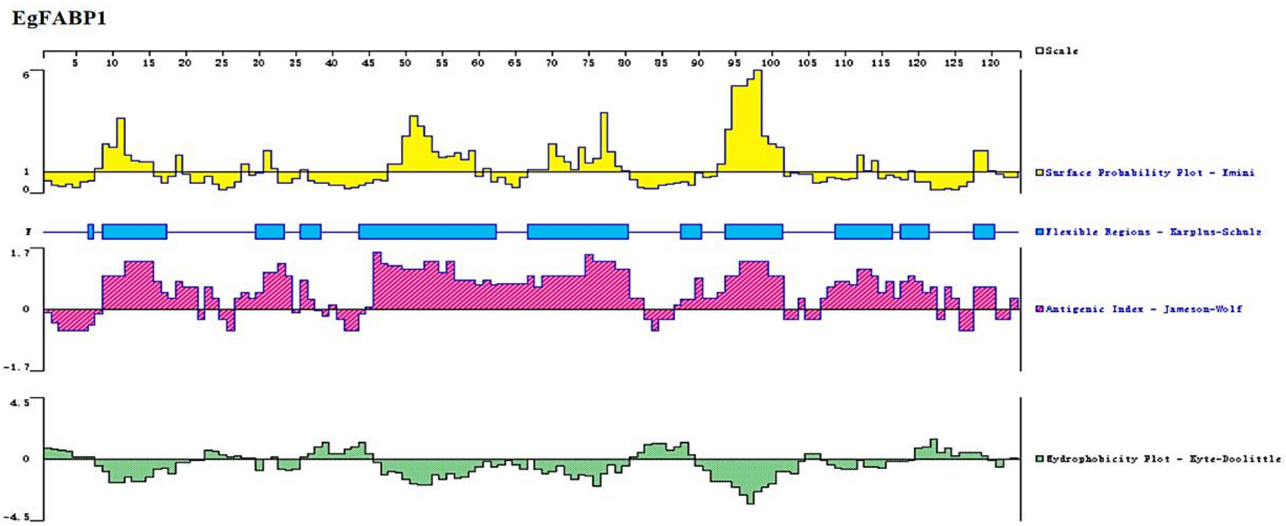

Supplementary Figure 6-2. The predicted B-cell epitopes of Protein EgTeg by DNASTAR. The yellow area represents Surface accessibility, the blue area represents Flexibility, the purple area represents Antigenicity and the green area represents Hydrophobicity. We need to obtain the regions above the threshold of the yellow area, the blue area and the purple area, and the regions below the threshold of the green area.

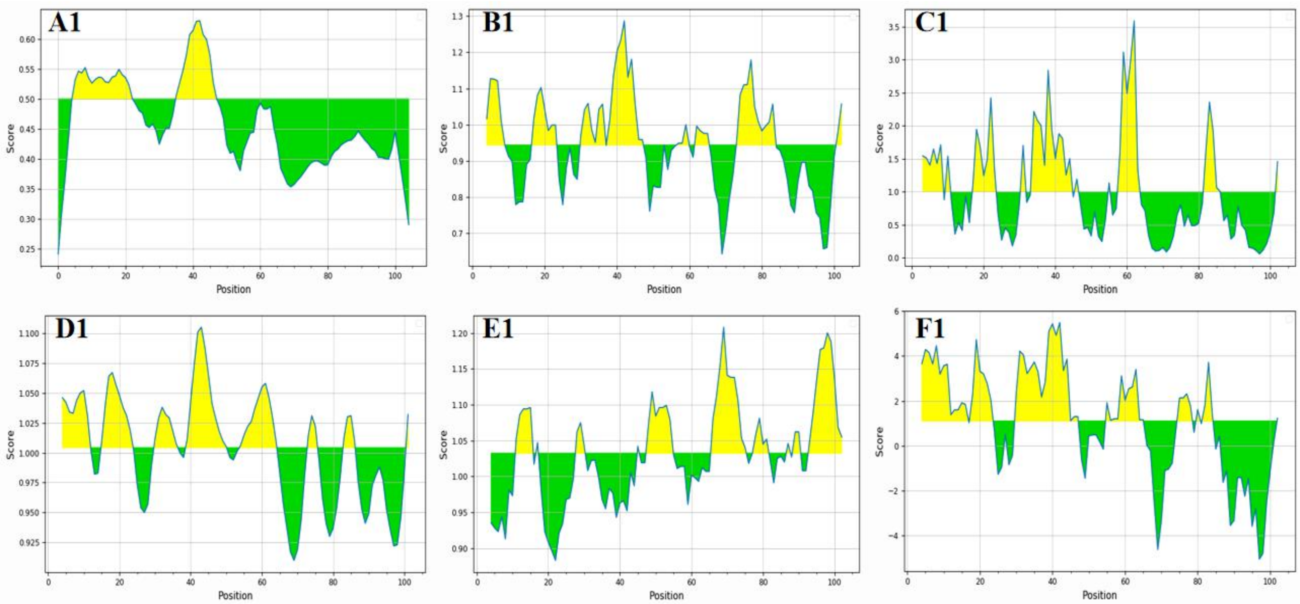

Supplementary Figure 7-1. The predicted B-cell epitopes of Protein EgTeg by IEDB. The A1 is Linear epitope, the B1 is β-turn, the C1 is Surface accessibility, the D1 is Flexibility, the E1 is Antigenicity and the F1 is Hydrophilicity. We need to obtain the yellow regions as the dominant result.

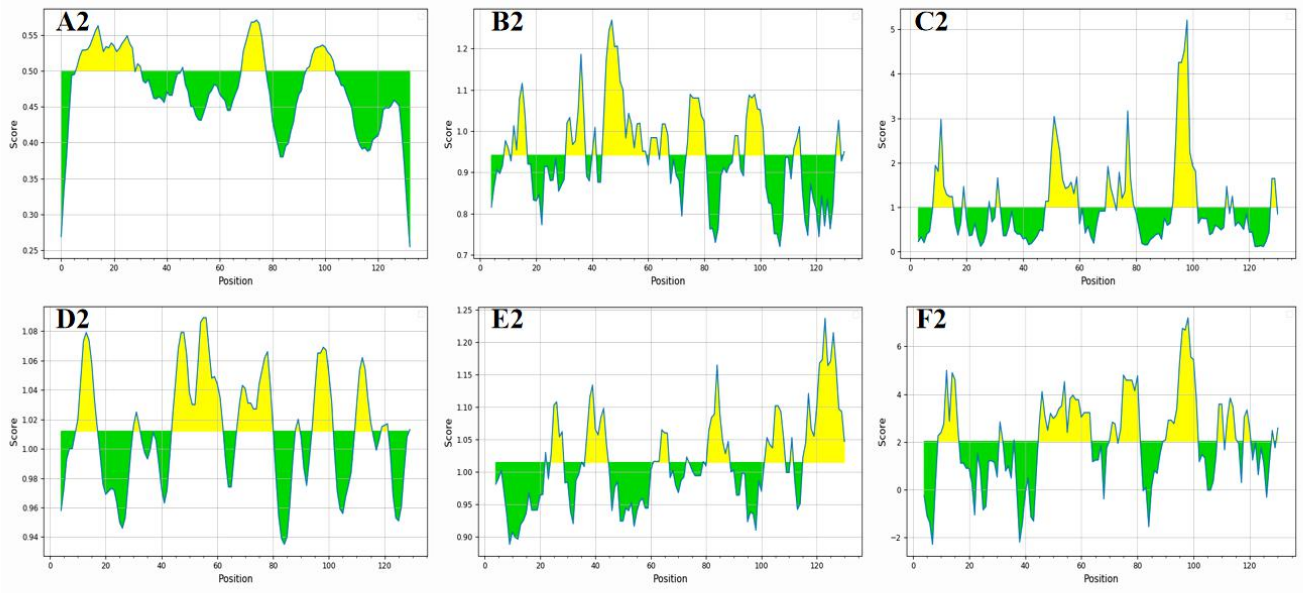

Supplementary Figure 7-2. The prediction of B-cell epitopes of Protein EgFABP1 by IEDB. The A2 is Linear epitope, the B2 is β-turn, the C2 is Surface accessibility, the D2 is Flexibility, the E2 is Antigenicity and the F2 is Hydrophilicity. We need to obtain the yellow regions as the dominant result.

Supplementary Table 4-1. The predicted B-cell epitopes of Protein EgTeg by DNASTAR and IEDB.

Software	Analysis Parameters	The areas of prediction of epitope
DNASTAR	Surface Accessibility	1-9 17-23 34-44 58-64 82-87 102-105
	Flexibility	5-13 15-25 30-37 39-51 53-65 72-76 83-87 100-103
	Antigenicity	1-25 30-51 57-66 82-87 102-105
	Hydrophilicity	1-9 16-21 31-46 56-64 80-89
IEDB	Surface Accessibility	4-12 16-24 30-48 58-65 82-87
	Flexibility	4-13 16-24 30-36 38-50 55-65 73-76 83-87
	Antigenicity	10-16 27-31 47-56 65-75 77-82 86-92 94-103
	Hydrophilicity	5-25 30-48 54-66 74-86

Supplementary Table 4-2. The predicted B-cell epitopes of Protein EgFABP1 by DNASTAR and IEDB.

Software	Analysis Parameters	The areas of prediction of epitope
DNASTAR	Surface Accessibility	7-16 47-60 70-80 93-102 128-131
	Antigenicity	9-21 27-35 46-83 87-102 107-123 128-131
	Flexibility	9-18 30-36 44-62 67-81 88-91 94-102 109-116 118-121 127-131
	Hydrophilicity	8-18 45-58 67-80 88-100
IEDB	Surface Accessibility	8-15 48-60 68-80 98-102 127-130
	Antigenicity	8-17 23-27 44-63 66-80 92-102 110-116
	Flexibility	24-29 35-46 62-67 80-89 101-108 115-133
	Hydrophilicity	10-16 45-63 70-81 107-113 116-120

3.9 The optimization of CD4⁺ T-cell epitopes

3.9.2 The docking between CD4⁺ T-cell epitopes and HLA-DRB1

Supplementary Table 5. The RDOCK results of all docking poses.

Pose	ZDOCK Score	E_vdw1	E_elec1	E_vdw 2	E_elec2	E_sol	E_RDock
Pose13	14.14	-71.7491	-7.10679	-76.4959	-7.83627	7.3	0.247355
Pose24	13.2	-63.0227	-0.0992405	-78.5563	1.51407	-6.7	-5.33734
Pose2	17.1	-91.4773	0.959933	-98.8716	-6.24189	1.1	-4.5177
Pose20	12.68	-67.3236	-2.90133	-77.5331	-2.78196	-0.1	-2.60376
Pose56	12.68	-62.5992	-1.04619	-72.1772	1.28832	-2.8	-1.64051

3.10 The construction of multi-epitope vaccine

The sequence of multi-epitope vaccine as follows:
MSRSEVEVLKKFERQFIIDQVDDTLKFERQLVTQLGRTLKQRYKFERQYTPERLFHFKGPSLSEVEVLKSDMPTEMKNFKFERQVWQVVILTGSYSKFERQYTPERLFHFKFGRHEYGAEALERAGSEVEVLKSDMPTEMKNFKFERQVWQVVILTGSYSKFERQYTPERLFHFKFGRGPSLTTMSRSEVGGSSGGKSDMPTEMKGGSSGGDDTLREYNADSSRPIGGSSGGLGRTLKQRYEGGPSLVDFVTRKMGNLVKKFERQFKLGEKFKKFERQLITVENGVMKGPSLFVTRKMGNLVKPKFERQKATVKVDEVVCVRTYHEYGAEALERAGFVTRKMGNLVKPKFERQKATVKVDEVVCVRTYGPSLKMEKSEGFGGSSGGLGGGKYKMRSESTFKGGSSGGKFKEVTPDSREVGGSSGGKHEQDDKTKV.
